# Denervation alters the secretome of myofibers and thereby affects muscle stem cell lineage progression and functionality

**DOI:** 10.1038/s41536-024-00353-3

**Published:** 2024-03-01

**Authors:** Henriette Henze, Sören S. Hüttner, Philipp Koch, Svenja C. Schüler, Marco Groth, Björn von Eyss, Julia von Maltzahn

**Affiliations:** 1 Leibniz Institute on Aging – Fritz Lipmann Institute, Beutenbergstrasse 11, 07745 Jena, Germany; 2 Faculty of Health Sciences Brandenburg, Brandenburg University of Technology Cottbus – Senftenberg, Universitätsplatz 1, 01968 Senftenberg, Germany

**Keywords:** Muscle stem cells, Regeneration

## Abstract

Skeletal muscle function crucially depends on innervation while repair of skeletal muscle relies on resident muscle stem cells (MuSCs). However, it is poorly understood how innervation affects MuSC properties and thereby regeneration of skeletal muscle. Here, we report that loss of innervation causes precocious activation of MuSCs concomitant with the expression of markers of myogenic differentiation. This aberrant activation of MuSCs after loss of innervation is accompanied by profound alterations on the mRNA and protein level. Combination of muscle injury with loss of innervation results in impaired regeneration of skeletal muscle including shifts in myogenic populations concomitant with delayed maturation of regenerating myofibers. We further demonstrate that loss of innervation leads to alterations in myofibers and their secretome, which then affect MuSC behavior. In particular, we identify an increased secretion of Osteopontin and transforming growth factor beta 1 (Tgfb1) by myofibers isolated from mice which had undergone sciatic nerve transection. The altered secretome results in the upregulation of early activating transcription factors, such as *Junb*, and their target genes in MuSCs. However, the combination of different secreted factors from myofibers after loss of innervation is required to cause the alterations observed in MuSCs after loss of innervation. These data demonstrate that loss of innervation first affects myofibers causing alterations in their secretome which then affect MuSCs underscoring the importance of proper innervation for MuSC functionality and regeneration of skeletal muscle.

## Introduction

Skeletal muscle is a highly organized tissue with a remarkable capacity to regenerate, which mainly originates from the tissue-resident muscle stem cells (MuSCs) that are characterized by the expression of the paired box transcription factor Pax7^1^. MuSCs are actively kept in a state of reversible quiescence through interactions with their niche. For example, myofiber-secreted Wnt4 as well as Oncostatin M are critical determinants for MuSC quiescence^2,3^. Upon skeletal muscle injury, MuSCs break quiescence, marked by a reduction of Pax7 levels concomitant with the rapid induction of the myogenic regulatory factors (MRFs) Myf5 and MyoD. Activated MuSCs can undergo several rounds of proliferation before MyoD induces the expression of Myogenin, causing withdrawal from the cell cycle and differentiation. Notably, a small subpopulation of myogenic progenitors undergoes self-renewal by returning to quiescence to replenish the stem cell pool^4^. Subsequently, the differentiated myocytes can fuse to each other to induce de novo myofiber formation or to damaged myofibers, ultimately leading to tissue repair. These newly formed myofibers can be identified by the high abundance of embryonic MHC (eMHC), encoded by *Myh3*, which is widely used as a readout for the regeneration status^5^.

The neuromuscular system represents the crossroad between peripheral nerves and skeletal muscle myofibers and is crucial for voluntary movements. During aging and in a number of neuromuscular pathologies, such as spinal muscular atrophy (SMA), motor neuron integrity, skeletal muscle innervation and signal transmission via the neuromuscular junction (NMJ) are impaired, which lead to a decline of skeletal muscle performance^6^. A newly described link between denervation and aging was recently demonstrated, as both aging and denervation by sciatic nerve transection caused a significant increase in the expression of the enzyme 15-prostaglandin dehydrogenase^7,8^. Inhibition of the enzyme activity restored functionality of the neuromuscular junction and increased motor neuron viability after denervation due to aging or peripheral nerve injury, representing a new potential therapeutic target.

Loss of skeletal muscle innervation affects myofibers as well as different resident cell populations, such as fibro-adipogenic progenitors (FAPs) and muscle-resident glia cells^9,10^. Additionally, MuSC numbers increase after loss of innervation concomitant with a higher amount of cytoplasm, increased motility and localization to the interstitial space^11,12^. Although MuSC numbers increase directly after denervation, long-term denervation leads to insufficient myogenesis, resulting in small myofibers with reduced contractile capacity, while their in vitro differentiation did not show significant alterations^13^. Whereas some studies identified a depletion of MuSCs after long-term denervation, others observed increased proliferation as well as normal regeneration and MuSC engraftment^12,14^. Interestingly, MuSCs can also actively participate in NMJ maintenance and their depletion induces NMJ degeneration and myofiber atrophy, underlining the importance of the bilateral cross talk between the peripheral nervous system and MuSCs^15,16^. Furthermore, MuSCs fuse into myofibers specifically in regions close to the NMJ after denervation, thereby contributing myonuclei, and supporting re-innervation^17^. However, it remains elusive whether MuSCs directly respond to denervation or whether changes in the niche cause alterations in MuSC functionality and gene expression.

Here, we demonstrate that the MuSC niche undergoes profound alterations after denervation, including changes in the myofiber secretome, which cause changes in MuSC behavior concomitant with changes in gene expression and protein abundance. Furthermore, we show that muscle regeneration is dependent on functional motor innervation, while the engraftment potential of MuSCs is independent of their innervation state. These results suggest that alterations in the niche environment drive the changes in MuSCs observed after denervation.

## Results

### Skeletal muscle denervation alters properties of MuSCs

**Fig. 1:**
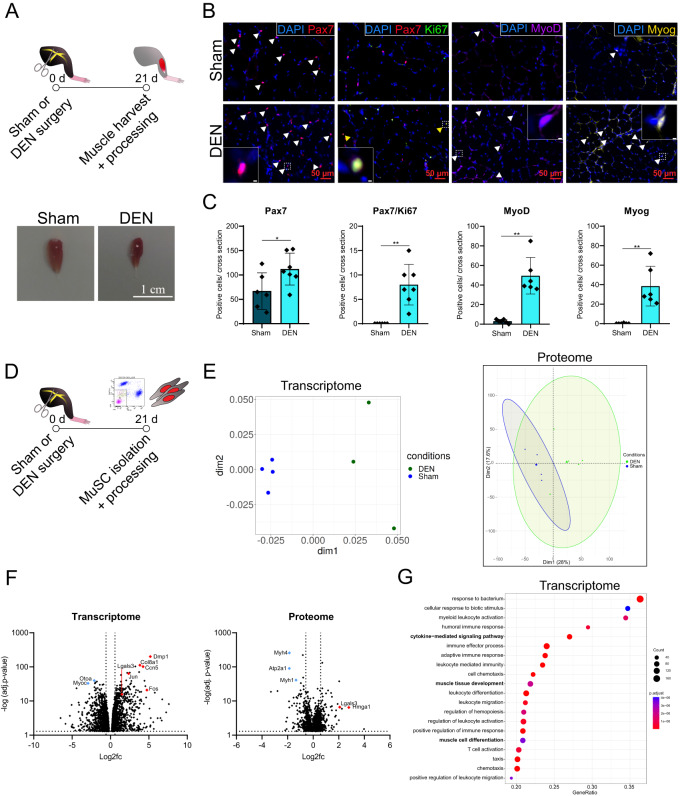
Skeletal muscle denervation alters properties of MuSCs. **A** Experimental scheme (upper panel). Male C57BL/6 J mice were subjected to either Sham or DEN surgery and TA muscles were harvested for histological analysis 21 days after surgery. Representative images of TA muscles of Sham or DEN operated mice 21 d after surgery (lower panel). Scale bar is 1 cm. **B** Representative images of immunofluorescent stainings of TA muscle cross sections 21 d after Sham or DEN surgery. White arrowheads indicate Pax7 positive cells (red), MyoD positive cells (purple) or Myog positive cells (yellow). Yellow arrowheads indicate Pax7/Ki67 double positive cells. Nuclei were counterstained with DAPI. Scale bar is 50 µm. Inserts shows individual cells positive for Pax7, Pax7/Ki67, MyoD or Myog. Scale bar for the inserts is 2 µm. **C** Quantifications of (B). *n* = 6 – 7 animals, each data point represents one animal. Statistical testing was done by unpaired two-tailed *t*-test with Welch’s correction. Error bars represent SD, *p < 0.05, **p < 0.01. **D** Experimental scheme. Male C57BL/6 J mice were subjected to either Sham or DEN surgery. 21 days after surgery, hind limb muscles were harvested for FACS-isolation of MuSCs. **E** Multidimensional scaling plot of transcriptomics (left) and principal component plot of proteomics (right) data of isolated MuSCs. Ellipses represent 95% confidence intervals. **F** Volcano plot of DEGs (left, adj. *p*–value < 0.05) and DAPs (right, adj. *p*–value < 0.05) in MuSCs of DEN mice. Represented is the log2 fold change relative to expression in Sham samples, with selected downregulated genes in blue and upregulated genes in red. Dotted lines mark log2 fold change of -/+ 0.58 and adj. *p*–value < 0.05. *n* = 3–4 animals for transcriptomics and *n* = 4–5 technical replicates for proteomics. **G** 20 most significant biological processes in MuSCs after denervation identified as activated via GSEA. GeneRatio represents the fraction of enriched genes within a GO term.

To identify the changes that occur in MuSCs after denervation, we first examined TA (*tibialis anterior*) cross sections from C57BL/6 J mice subjected to control (Sham) or denervation (DEN) surgery 7, 21 or 42 days prior to isolation, allowing us to choose the appropriate time point for in-depth analysis of MuSCs (Supplementary Fig. 1A–G). Briefly, at 21 d after denervation the following parameters were very pronounced: muscle weight loss driven by myofiber atrophy without loss of myofibers, as well as MuSC proliferation evidenced by a sharp increase in the number of Pax7/Ki67 positive cells. Therefore, we chose this time point for further analyses (Fig. 1A). Notably, normalizing mononucleated marker positive cells to muscle area—as it is often done—is not applicable here due to the strong size difference between muscle cross sections of surgery groups. Therefore, cell population counts are represented as absolute numbers of mononucleated marker positive cells per cross section throughout the manuscript. In addition to an increase in proliferation (Pax7/Ki67) specifically in mainly fast-twitch muscles (TA and Gastrocnemius, Supplementary Fig. 1 H, I), we identified a commitment to the myogenic lineage (MyoD/Myog) (Fig. 1B, C). Of note, intrinsic differences of slow and fast muscles, such as a higher number of MuSCs on type I compared to type II myofibers, indicated inherent MuSC differences and might explain the differences observed here^18,19^. This commitment was accompanied by an increase in the expression of eMHC in all muscles after DEN, with a peak at 7 d post-surgery in TA muscles (Supplementary Fig. 1J–N) and a shift in myofiber types (Supplementary Fig. 1O–T), suggesting a deregulation of myosin isoform expression in denervated TA muscles.

Next, we asked which specific changes occur in MuSCs after denervation, comparing MuSCs 21 days after Sham or DEN surgery (Fig. 1D and Supplementary Fig. 2A, B). MuSCs were clearly distinguishable according to the surgery group on both the mRNA and protein level, suggesting profound alterations after denervation (Fig. 1E). In the transcriptome, 2613 DEGs were detected (adj. *p*-value < 0.05) while the proteome analysis revealed 1096 differentially abundant proteins (DAPs, *q*-value < 0.05) between surgery groups (Fig. 1F). As *Lgals3* (encoding Galectin-3) was significantly upregulated after DEN both on the transcriptome and proteome level, we used it to exemplarily validate the omics data (Supplementary Fig. 2C). However, another validation of the transcriptome data is given for the expression analysis of *Junb* (Fig. 5B and Supplementary Fig. 7A). GSEA of the transcriptome identified the activation of “muscle tissue development” and “muscle cell differentiation”, indicating changes in MuSCs reminiscent of regeneration following denervation (Fig. 1G). Notably, we also identified the activation of immune response-linked processes (“cytokine-mediated signaling pathway”) suggesting an inflammation-associated milieu in the MuSC niche. Strikingly, an overrepresentation analysis (ORA) with an additional filter for log2 fold changes of >0.5, translating to a change of +1 (upregulated genes only), indicated that genes upregulated in MuSCs after DEN are involved in processes like “muscle tissue development”, “muscle cell differentiation” or “axon development” (Supplementary Fig. 2D). These results strengthen our notion that denervation induces regeneration-like gene expression changes in MuSCs. With a correlation analysis between DEGs and DAPs that were additionally filtered for log2 fold changes of </> −0.58/ + 0.58 (translating to a change of ± 1.5) we show that a subset of genes, including transcription factors such as Jun and Fos as well as transmembrane receptors like Integrins (Itgb4 and Itga6), is similarly regulated at the mRNA and protein level (Supplementary Fig. 2E, F), suggesting a correlation between changes of the MuSC transcriptome and proteome after denervation. In a study by Machado et al.^20^, the transcriptomes of in situ fixed isolated MuSCs and MuSCs in early activation were compared. Among the genes that are highly expressed in quiescent MuSCs but downregulated during activation are *Abat*, *Dhcr24*, *Mapt*, *Mccc2* and *Prodh*, which we also found to be downregulated in MuSCs after DEN. Since these genes seem to be important for MuSC quiescence, their downregulation might cause the premature activation and break of quiescence observed in MuSCs after denervation. Of note, the unique factors that did not overlap between the analyses include genes of all classes, such as genes encoding transcription factors, receptors or metabolic enzymes.

### Alterations in the myofiber niche are driving MuSC dysfunction in denervated skeletal muscle

**Fig. 2:**
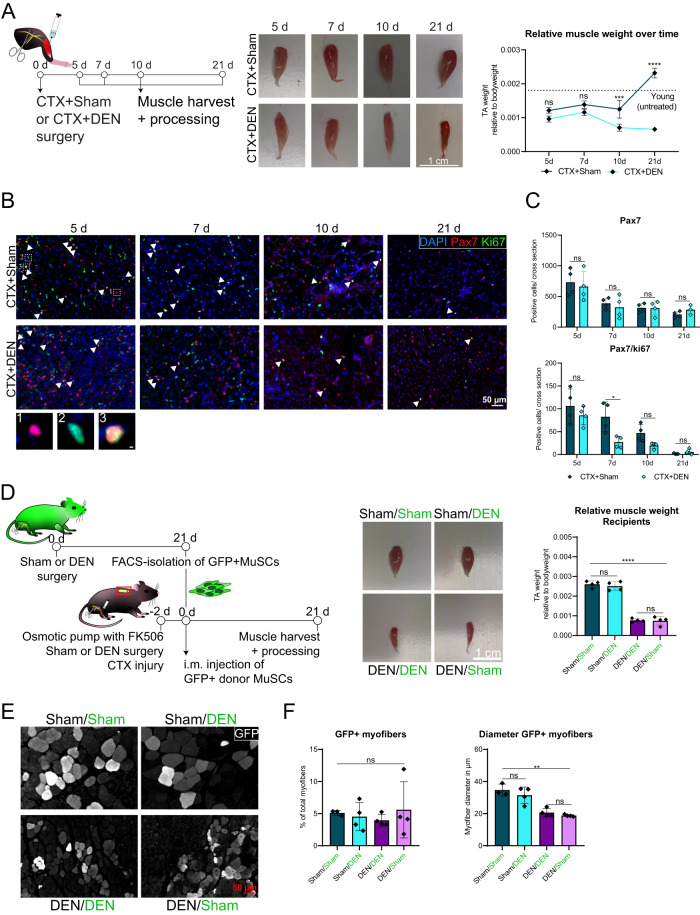
Alterations in the myofiber niche are driving MuSC dysfunction in denervated skeletal muscle. **A** Experimental scheme (left). Male C57BL/6 J mice were subjected to cardiotoxin (CTX) – induced muscle injury in combination with either Sham or DEN surgery and TA muscles were harvested for histological analysis at the respective time points. Representative images of TA muscle of CTX+Sham or CTX + DEN operated mice at each time point after surgery (middle). Scale bar is 1 cm. TA muscle weight relative to bodyweight at each time point after surgery (right). The dotted line represents the average TA muscle weight relative to body weight of age-matched control mice. *n* = 16 for control mice and *n* = 4 for Sham and DEN mice. **B** Immunofluorescent staining of TA muscle cross sections of CTX+Sham and CTX + DEN operated mice at different time points after surgery. MuSCs are marked by Pax7 (red), proliferating cells are marked by Ki67 (green) and nuclei are counterstained with DAPI (blue). White arrowheads indicate Pax7/Ki67 double positive cells. Scale bar is 50 µm. Panel of inserts shows individual cells positive for Pax7 (1, red), Ki67 (2, green) or Pax7 and Ki67 (3, merge). Scale bar for the inserts is 2 µm. **C** Quantification of (**B**). *n* = 4 animals per surgery group at each time point, each data point represents one animal. Statistical testing was done by Two-way-ANOVA with post-hoc Tukey’s multiple comparisons test. Error bars represent SD. ns = not significant, *p < 0.05, ***p < 0.001, ****p < 0.0001. **D** Experimental scheme (left). Male and female CAG-GFP mice were subjected to Sham or DEN surgery. 21 days after surgery, hind limb muscles were harvested for FACS-isolation of GFP positive donor MuSCs. Male C57BL/6 J recipient mice obtained CTX injury, implantation of an osmotic pump containing the immunosuppressive FK506 and either Sham or DEN surgery 2 days before intramuscular injection of ten thousand donor MuSCs. After 21 days, TA muscles were harvested for histological analyses. Representative images of TA muscles of each group (middle; donor is indicated in green). Scale bar is 1 cm. TA muscle weight relative to bodyweight for each group at 21 days after MuSC transplantation (right). **E** Immunofluorescent staining of TA muscle cross sections for GFP 21 d after MuSC transplantation. Scale bar is 50 µm. **F** Quantification of (**E**) with GFP positive myofibers as percentage of total myofibers (left) and myofiber diameter of GFP positive myofibers (right). *n* = 3–4 animals per surgery group, each data point represents one animal. Statistical testing was done by One-way-ANOVA with post-hoc Tukey’s multiple comparisons test. Error bars represent SD. ns not significant, **p < 0.01, ****p < 0.0001.

To assess whether the observed changes in MuSCs are translated into functional impairments, we investigated the regeneration of skeletal muscle concomitant with denervation, allowing us to analyze whether regeneration is directly depending on innervation (Fig. 2A). CTX + DEN muscles had a constantly lower relative muscle weight compared to CTX + Sham muscles, mainly due to strong myofiber atrophy, thereby mimicking conditions when acute trauma of leg muscles coincide with lesions of the spinal cord (Fig. 2A and Supplementary Fig. 3A, B). Interestingly, 21 d after surgery regenerating myofibers of CTX + DEN muscles contained less nuclei than control muscles (Supplementary Fig. 3C). However, this was not observed at an earlier time point of regeneration (10 d post-surgery) and thus, it is likely that the size reduction of regenerating myofibers in CTX + DEN muscles is not only driven by a smaller number of nuclei fusing into myofibers but additionally by a decline in protein synthesis. Moreover, we identified an increased amount of eMHC positive myofibers in CTX + DEN muscles in the late phase of regeneration (Supplementary Fig. 3D). This suggests that, while the formation of new myofibers by MuSC fusion during early regeneration seemed to be independent of innervation (same percentage of eMHC positive myofibers between surgery groups at 5 and 7 days post injury), the downregulation of eMHC during late regeneration is dependent on the neuronal input. Reinnervation of skeletal muscle typically takes place around day 8 after injury supporting our notion that reinnervation plays an important role in MuSC behavior as well as formation and maturation of myofibers as observed here^21,22^. This deregulation of regeneration dynamics was accompanied by a higher proportion of fibrotic tissue in CTX + DEN muscles, indicating inadequate tissue regeneration (Supplementary Fig. 3G, H).

Surprisingly, the total number of Pax7 positive MuSCs per cross section was comparable between surgery groups throughout the regeneration time course, while proliferation of MuSCs was reduced in CTX + DEN muscles until day 10 after injury (Fig. 2B, C). However, denervated muscles were considerably smaller than their Sham counterparts and therefore display a higher density of Pax7 positive MuSCs. Notably, the expression of the differentiation marker Myogenin showed similar dynamics as Ki67 in MuSCs (Supplementary Fig. 3E, F).

We then asked whether the phenotypic alterations in MuSCs following denervation persist in a fully innervated context or whether changes in the niche triggered by denervation are causing those alterations. Therefore, MuSCs from CAG-GFP mice^23^, which are characterized by ubiquitous GFP expression, after Sham or DEN surgery were transplanted into CTX-injured TA muscles of Sham or DEN operated C57BL/6 J recipient mice (Fig. 2D; MuSC donors are highlighted in green). Although DEN surgery induced a strong myofiber atrophy in the TA muscles of donor and recipient mice, the innervation state of donor MuSCs did not influence the recipient muscle weight (Fig. 2D–F and Supplementary Fig. 3I, J). Interestingly, the percentage of GFP positive myofibers, which originate from either the fusion of donor-derived MuSCs to each other or with resident MuSCs from the recipient or to existing myofibers, was comparable between the four surgery groups, suggesting that denervation does not affect the ability of MuSCs to fuse (Fig. 2E, F). Even though the increased number of Pax7 positive MuSCs in muscles that received MuSCs from a denervated donor did not lead to an increased proportion of GFP positive myofibers (Supplementary Fig. 3K), the size of GFP positive myofibers was severely decreased in DEN recipient muscles independent of the innervation state of the MuSC donor (Fig. 2F). This indicates that the MuSC niche is strongly affected by denervation and is causing the main effects on MuSCs following denervation reminiscent of the activation and differential gene expression we observed.

### Denervation disrupts the immediate MuSC niche

**Fig. 3:**
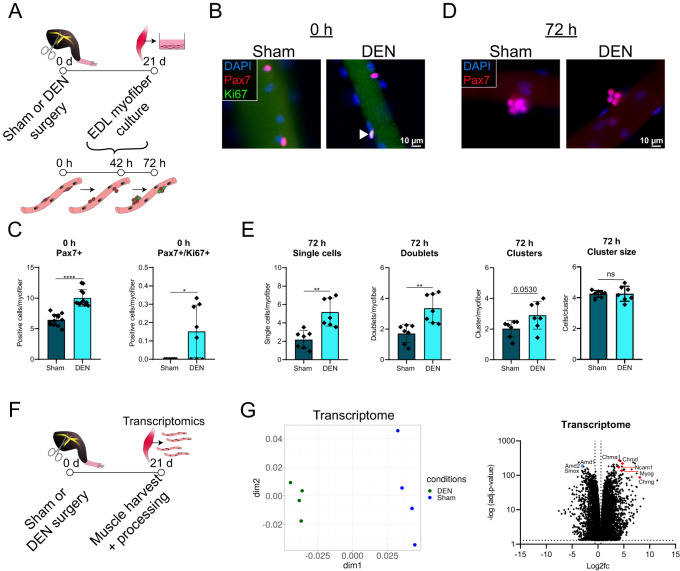
Denervation disrupts the immediate MuSC niche. **A** Experimental scheme. Male C57BL/6 J mice were subjected to either Sham or DEN surgery and EDL muscles were harvested for myofiber culture 21 days later. **B** Immunofluorescent staining of isolated myofibers for Pax7 (red) and Ki67 (green) 0 h after isolation. White arrowhead indicates Pax7/Ki67 double positive cell. Nuclei were counterstained with DAPI. *n* = 12 animals per surgery group, each data point represents one animal. Scale bar is 10 µm. **C** Quantification of (**B**). **D** Immunofluorescent staining of isolated myofibers for Pax7 (red) after 72 h of culture. Nuclei were counterstained with DAPI. Scale bar is 10 µm. **E** Quantification of (**D**). *n* = 7 animals per surgery group, each data point represents one animal. Statistical testing was done by unpaired two-tailed *t*-test with Welch’s correction. Error bars represent SD. ns = not significant, *p < 0.05, **p < 0.01, ****p < 0.0001. **F** Experimental scheme. Male C57BL/6 J mice were subjected to either Sham or DEN surgery. 21 days later, EDL muscles were harvested and single myofibers were isolated for transcriptomic analysis. **G** Multidimensional scaling plot of myofiber transcriptome (left). Volcano plot of DEGs (adj. *p*–value < 0.05) in myofibers of DEN mice (right). *n* = 4 animals per surgery group, each data point represents one animal. Represented is the log2 fold change relative to expression in Sham samples, with selected downregulated genes in blue and upregulated genes in red. Dotted lines mark log2 fold change of -/+ 0.58 and adj. *p*–value < 0.05.

The previous experiments suggest that the myofiber niche is driving MuSC dysfunction after denervation. Therefore, we wondered how MuSCs behave when cultured on their adjacent myofibers isolated from mice after Sham or DEN surgery (Fig. 3A). In line with the observed increase in Pax7 positive and Pax7/Ki67 positive cells in DEN TA cross sections (Fig. 1C), we observed an increase in the numbers of those cell populations on EDL myofibers after denervation (Fig. 3B, C). After 42 and 72 h of culture, myofibers from DEN mice harbored clearly more single cells, doublets and clusters per myofiber (Fig. 3D, E, Supplementary Fig. 4A–C). Although MuSCs on DEN myofibers were in a more proliferative state directly after isolation, this did not lead to an increase in cluster size at any time point (Fig. 3E and Supplementary Fig. 4D), indicating that proliferation was either only slightly or not permanently increased. Interestingly, activation of MuSCs was not affected in this assay (Supplementary Fig. 4E). Together, these results strengthen the notion that changes in MuSCs after denervation are driven by the immediate niche.

Since myofibers comprise the largest component of the immediate MuSC niche, we investigated changes in the transcriptome of myofibers following denervation (Fig. 3F). We observed a clear clustering of surgery groups, driven by massive changes in the expression landscape after denervation, including upregulation of genes associated with an inflammatory response (Fig. 3G and Supplementary Fig. 5A). Notably, the transcriptome of whole muscles containing different cell types following denervation and myofibers that were isolated by collagenase digestion to remove contaminating cell types shared over 4000 DEGs, with 99% of genes being regulated in the same direction (Supplementary Fig. 5B–F), emphasizing that myofibers display major changes following denervation.

### Factors secreted by myofibers drive MuSC fate alterations after denervation

**Fig. 4:**
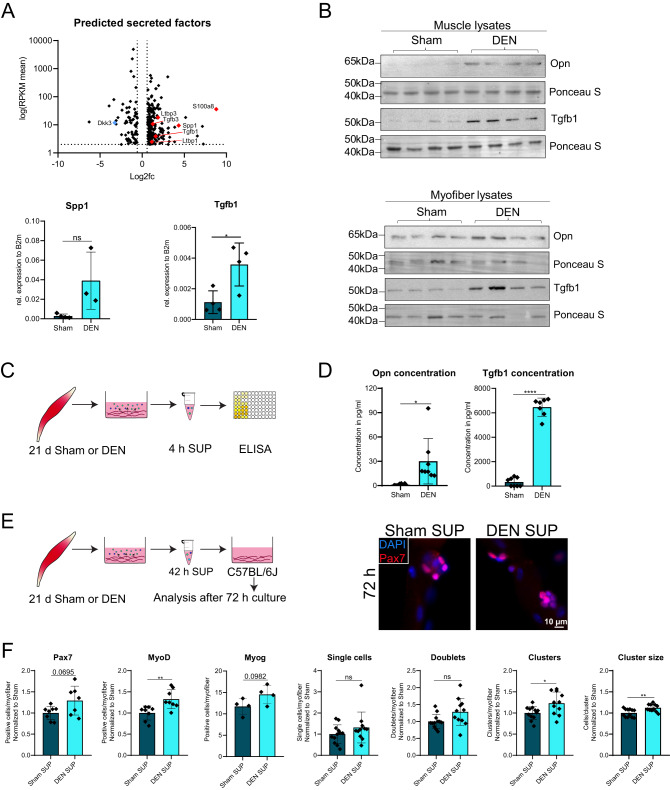
Factors secreted by myofibers drive MuSC fate alterations after denervation. **A** Volcano plot of gene expression changes of predicted secreted factors in the denervated myofiber transcriptome (upper graph). Prediction of secreted factors was performed with the DAVID bioinformatics database. Dotted lines mark log2 fold change of -/+ 0.58 and average RPKM of 2. qRT-PCR for *Spp1* and *Tgfb1* mRNA in myofibers of Sham or DEN operated mice (lower graphs). *n* = 3–4 animals per surgery group, each data point represents one animal. **B** Western blot analysis of Opn and Tgfb1 protein level in muscle lysates (upper panel) and myofiber lysates (lower panel) of Sham or DEN operated mice. **C** Experimental scheme. Male C57BL/6 J mice were subjected to either Sham or DEN surgery and EDL muscles were harvested for myofiber culture 21 days later. After 4 h of culture, myofiber supernatant was collected for ELISA. **D** Opn and Tgfb1 concentrations in the myofiber supernatant samples after 4 h of culture. Calculation of standard curve and concentrations was performed using an online ELISA calculator (https://www.arigobio.com/ELISA-calculator). Each sample was analyzed in duplicates. *n* = 7–8 animals per surgery group, each data point represents one animal. **E** Experimental scheme (left). Male C57BL/6 J mice were subjected to either Sham or DEN surgery and EDL muscles were harvested for myofiber culture 21 days later. After 42 h of culture, myofiber supernatant was collected and directly used for treatment of freshly isolated myofibers from healthy C57BL/6 J mice. Immunofluorescent staining of isolated myofibers for Pax7 (red) 72 h after isolation and culture in Sham or DEN supernatant (SUP) (right). Nuclei were counterstained with DAPI. Scale bar is 10 µm. **F** Quantification of (**E**, right). Plots show data normalized to Sham samples. *n* = 4–12 animals per surgery/treatment group, each data point represents one animal. Statistical testing was done by unpaired two-tailed *t*-test with Welch’s correction. Error bars represent SD. ns = not significant, *p < 0.05, **p < 0.01, ****p < 0.0001.

We speculated that alterations in factors secreted by myofibers cause the changes in MuSC functionality following denervation. Therefore, we performed a DAVID-based prediction analysis of the myofiber transcriptome for secreted/extracellular factors. Thereby, we obtained a list of 691 predicted secreted factors, which we filtered for an average RPKM value of at least 2 between Sham and DEN samples. Since the RPKM value indicates the level of gene expression we used it here to identify genes which display a reasonably high expression in both surgery conditions. From the remaining 272 predicted secreted factors (Fig. 4A), we chose *Spp1* (secreted phosphoprotein 1, encoding the protein Opn) and *Tgfb1* (transforming growth factor beta 1) for further analyses due to their central role in inflammation and cell fate specification. Moreover, *S100a8*, which was shown before to be involved in the early inflammatory response after peripheral nerve injury, was strongly upregulated in myofibers after denervation, indicating an ongoing immune reaction^24^. We identified an increased gene expression of *Spp1* and *Tgfb1* in myofibers after denervation and a strongly increased protein abundance of Opn and Tgfb1 in whole muscle as well as myofibers following denervation (Fig. 4A, B). Strikingly, ELISA analysis of supernatants from cultured myofibers from Sham or DEN muscles corroborated the prediction analysis, as we detected a significant accumulation of both proteins in the supernatant of myofibers from mice that had undergone DEN surgery (Fig. 4C, D). To investigate whether Opn and Tgfb1 are drivers of MuSC activation after DEN, we isolated myofibers with their adjacent MuSCs from C57BL6/J mice and treated them with Opn (for 72 h) or Tgfb1 (for 48 h) recombinant proteins to mimic the high protein abundance in the supernatant of DEN myofibers (Supplementary Fig. 6A–F). Surprisingly, neither Opn nor Tgfb1 alone caused alterations in the number of single cells, clusters or cluster size, indicating that a single factor is not sufficient to induce DEN-like phenotypes in myofiber-associated MuSCs. Therefore, we next examined whether the whole myofiber secretome is sufficient to induce MuSC proliferation, as seen directly after isolation of myofibers from DEN mice (Fig. 3C). In brief, we added culture supernatants from myofibers of Sham or DEN mice to cultures of isolated myofibers with their adjacent MuSCs from healthy C57BL/6 J mice (Fig. 4E). Strikingly, the supernatant (SUP) from DEN myofibers was sufficient to increase the number of Pax7 positive, MyoD positive and Myog positive cells per myofiber as well as the number of clusters and the cluster size (Fig. 4F). Thereby, we demonstrate that changes in the myofiber secretome cause MuSC expansion, further supporting our notion that the altered microenvironment following denervation is driving MuSC alterations. We then investigated whether MuSC activation by the secretome of DEN myofibers could be blocked by neutralizing Tgfb1 from the supernatant (SUP). To this end, isolated myofibers from C57BL6/J mice were incubated with supernatant (SUP) from Sham or DEN myofibers together with a blocking antibody targeting Tgfb1 (Supplementary Fig. 6G–I). Interestingly, blocking of Tgfb1 was not sufficient to prevent the increased cluster formation induced by SUP from DEN myofibers, further strengthening the notion that one factor alone is not sufficient to drive MuSC activation after denervation. Therefore, we next used a cocktail of recombinant proteins (Opn, Chrd and Ostn), all of which were found among the upregulated predicted secreted factors (Supplementary Fig. 6K–M). Surprisingly, incubation of isolated myofibers with their adjacent MuSCs with the recombinant protein cocktail even decreased the number of single cells and clusters per myofiber, opposite to the effects from the DEN SUP. We suggest that the right combination of all or the majority of secreted factors in the supernatant from DEN conditions are required to cause the alterations in MuSCs – at least in the 72 h time frame which was analyzed here.

### Denervation induces expression of *Junb* in MuSCs

**Fig. 5:**
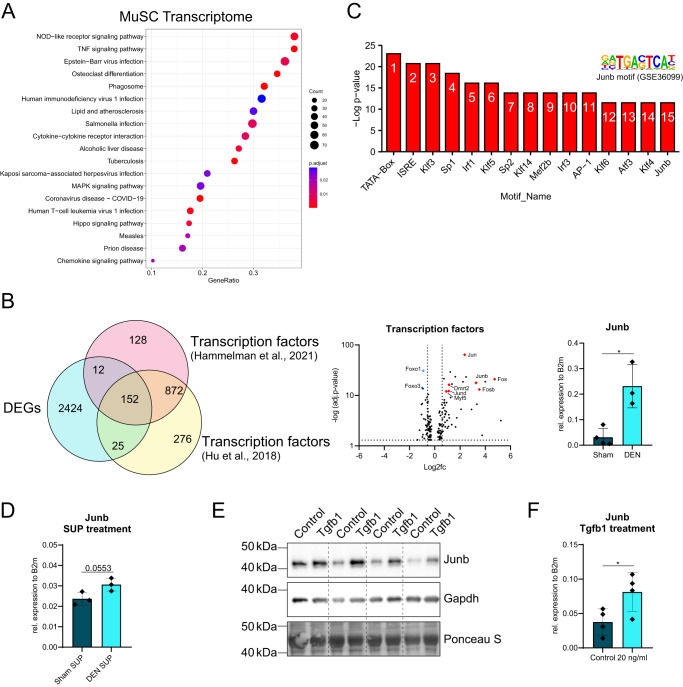
Denervation induces expression of *Junb* in MuSCs. **A** 20 most significant KEGG pathways in MuSCs after denervation identified as activated via GSEA. GeneRatio represents the fraction of enriched genes within a KEGG pathway. **B** Overlap analysis of DEGs from MuSC transcriptome (blue circle) and publicly available lists of mouse transcription factors (red and yellow circle) (left) and Volcano plot of the 152 identified transcription factors in the intersection (middle). Represented is the log2 fold change relative to expression in Sham samples, with selected downregulated genes in blue and upregulated genes in red. Dotted lines mark log2 fold change of -/+ 0.58 and adj. *p*–value < 0.05. qRT-PCR for *Junb* mRNA in MuSCs of Sham or DEN operated mice (right). **C** HOMER known motif analysis showing top 15 motif enrichments in denervated MuSCs. **D** qRT-PCR for *Junb* mRNA in myoblasts that were treated for 6 h with supernatant (SUP) of myofibers from Sham or DEN operated mice. **E** Western blot analysis of Junb protein level in lysates of myoblasts cultured under growth conditions. Myoblasts were treated either with 20 ng/ml Tgfb1 recombinant protein or solvent control (0.1% BSA in 10 mM citric acid). **F** qRT-PCR for *Junb* mRNA in cultured myoblasts after treatment with Tgfb1 recombinant protein. *n* = 3–4 animals per surgery/treatment group, each data point represents one biological replicate. Statistical testing was done by unpaired two-tailed *t*-test with Welch’s correction. Error bars represent SD. (*) *p* < 0.05.

After demonstrating that alterations in the myofiber secretome by denervation affect MuSC behavior (Fig. 4), we asked whether myofiber-secreted factors directly affect the expression of target genes in MuSCs. Therefore, we first performed a KEGG pathway enrichment analysis of the MuSC transcriptome and identified pathways activated in MuSCs following denervation, like “MAPK signaling pathway” and “Hippo signaling pathway” among the top activated pathways, both being important for cell proliferation (Fig. 5A). To identify potential transcriptional regulators we compared the DEGs of the MuSC data set with two publicly available lists of murine transcription factors^25,26^, resulting in an overlap of 152 differentially expressed transcription factors (Fig. 5B). Of those, the gene expression of *Jun* and *Fos* family members was increased in MuSCs after denervation. Interestingly, only *Junb* motifs were also found in the top 15 enriched motifs in a HOMER known motif analysis (Fig. 5C). Additionally, we observed a very pronounced upregulation of *Junb* expression in MuSCs after denervation (Fig. 5B, right and Supplementary Fig. 7A). To investigate whether induction of *Junb* expression in MuSCs from denervated mice was driven by myofiber-secreted factors, we incubated myoblasts of healthy C57Bl/6 J mice with supernatants from isolated myofibers of Sham or DEN mice. 6 h later, we assessed *Junb* gene expression and detected a clear trend for increased *Junb* expression in myoblasts incubated with supernatant from denervated myofibers (Fig. 5D). To further investigate the role of *Junb* in MuSCs after denervation, we used a publicly available list of Junb target genes^27^ and compared them to significantly upregulated genes (adj. *p*–value < 0.05, log2fc > 0.5) from our DEN MuSCs transcriptome (Supplementary Fig. 7B). From the 702 DEGs, 409 are Junb target genes, accounting for ~58% of DEGs. By performing ORA and generating enrichment plots of Junb over non-Junb target genes, we identified processes in DEN MuSCs where Junb target genes are involved (Supplementary Fig. 7C). Interestingly, we observed an enrichment of processes like “response to interferon-alpha” and “response to interferon-beta”, which is in line with our GSEA from DEN MuSCs (Fig. 1G), indicating a response to inflammatory signals.

**Fig. 6:**
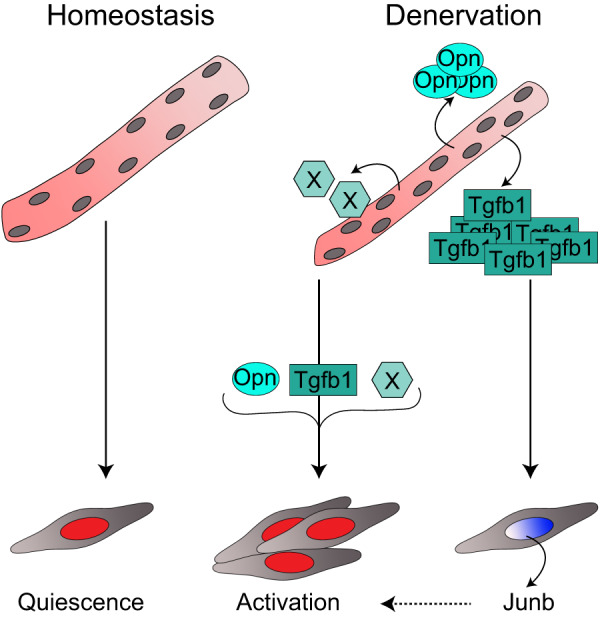
Graphical summary. Skeletal muscle denervation is driving MuSCs activation by myofiber-secreted factors.

As Tgfb1 was strongly induced in myofibers after denervation (Fig. 4A and Supplementary Fig. 7D) and accumulated in the supernatant of myofibers from DEN mice (Fig. 4D), we tested whether Tgfb1 alone was able to induce *Junb* expression in myoblasts (Fig. 5E, F). Although it has been shown before that Tgfb can induce *Junb* expression, for example during breast cancer invasion^28^, it is unknown whether *Junb* in MuSCs is a target gene of myofiber-secreted Tgfb1 in denervated muscle. Strikingly, *Junb* expression and protein abundance in MuSCs were increased after incubation with Tgfb1, indicating that Tgfb1 is acting as one of the upstream regulators of Junb (Fig. 5E, F). Together, we demonstrate here that denervation alters the myofiber secretome, which then drives gene expression changes in MuSCs, leading to altered functionality (Fig. 6).

## Discussion

Here, we show an increase in the number of MuSCs in total and specifically proliferating MuSCs with a peak at 21 d after denervation. Furthermore, we demonstrate that the myofiber secretome changes after denervation, which then causes MuSCs to undergo profound alterations, ultimately leading to an impaired regenerative capacity. Of note, we suggest that the alterations in MuSCs are dynamically regulated since MuSCs contributed similarly to myofibers independent of the innervation status of the donor or the recipient. Moreover, we detected an increased secretion of Tgfb1 from myofibers after denervation surgery, inducing the expression of *Junb* in myogenic cells.

While we identified an increase in the number of MuSCs after 21 days of denervation, we speculated that MuSC alterations after denervation are – to some extent – driven by modifications in the stem cell niche. Along these lines, snRNAseq analyses demonstrated that denervation causes a dramatic decline of ligand-receptor interactions between myofibers and muscle resident cells such as MuSCs, leading to an overall reduction of intercellular communication in denervated muscles^29^.

We demonstrate that factors released from myofibers after denervation cause MuSC alterations on the gene expression level and myogenic commitment. We identified an increased secretion of Tgfb1 from myofibers after denervation, causing enhanced expression of *Junb* in myogenic cells. Of note, Tgfb1, a secreted cytokine, plays a central role in inflammation and fibrosis, especially during tissue regeneration^30^, phenotypes we observed in muscles after denervation and which were reported previously^31^. Interestingly, inhibition of the enzyme 15-PGDH, a contributing factor to muscular atrophy during aging and after sciatic nerve transection, caused a reduction in the Tgfb-signaling pathway concomitant with a decrease in the expression of several key components of the pathway^7^. Strikingly, 15-PGDH gene expression was also significantly increased in our data set of denervated myofibers compared to control (data not shown) suggesting a link between increased 15-PGDH expression and Tgfb1 secretion by myofibers after denervation contributing to myofiber atrophy.

Further analyses of the transcriptome of MuSCs after denervation revealed, in addition to the strong induction of *Junb* gene expression, an activation of the MAPK signaling pathway and an enrichment of Junb motifs, which is in line with recent transcriptome analyses that indicated an activation of the MAPK signaling pathway and an upregulation of *Junb* mRNA in TA muscle after denervation^29,32^. Lin et al. speculate that Fgf13, which is secreted by myofibers and significantly upregulated in denervated myofibers both in their data set and in the data presented here, could activate MAPK signaling, as the MAPK pathway is a known downstream pathway of the FGF family. However, although Lin et al. observed an activation of the MAPK signaling pathway in MuSCs and an increase of *Fgf13* gene expression in myofibers after denervation, they did not detect major changes in MuSC numbers as we describe here. These discrepancies might arise from experimental differences like time points analyzed (14 vs 21 d after DEN), differences in the isolation protocol (nuclei vs FACS-purified MuSCs) or analysis method (snRNAseq with bioinformatic analyses vs. immunofluorescence stainings and bulk RNAseq)^29^.

*Junb* is one of the downstream effector genes of MAPK signaling, which can be activated by Tgfb and Bmp2 while *Junb* overexpression alone is sufficient to impair myogenic differentiation^30,33,34^. However, it was also shown that *Junb* is involved in early MuSC activation after injury, inducing the regenerative response. Interestingly, when comparing the signatures of MuSCs after denervation with the ones of MuSCs after injury of skeletal muscle, we found that in both cases *Junb* is upregulated. A study by Almada et al. described that *FOS* and other transcription factors from the AP1-family (including *Junb*) were rapidly induced within hours after muscle damage^35^. Along those lines, *Junb* expression was shown to be upregulated as early as 0.5 h after muscle injury and returning to baseline by 32 hpi, indicating its involvement in early MuSC activation and proliferation in the regenerative response^36^.

Moreover, it was described that *FOS*-expressing MuSCs are primed for a fast induction of proliferation and thereby muscle regeneration responses. Additionally, the direct *FOS*-target gene *Art1* was determined as a possible mediator of the very early transition from quiescence to activation. Interestingly, *Art1* was also significantly upregulated in our data set of MuSCs after denervation in addition to the increased gene expression of *Fos* and *Junb* (data not shown). Therefore, MuSCs from DEN muscles might express constantly high levels of *Junb*, inducing activation of MuSCs via *Art1* but blocking functional myogenic differentiation, causing an impaired response.

Although Tgfb1 was shown to inhibit myoblast fusion and differentiation, the effect of Tgfb1 on myoblast proliferation seems to be context dependent^30,37,38^. Reduction of the aberrant *Junb* expression in MuSCs or reduction of Tgfb1 levels in denervated muscles might be an additional starting point for improving MuSC functionality and thereby whole muscle health after loss of innervation. In addition to Tgfb1, other trophic factors are secreted by myofibers after denervation, such as Opn, similar to recent studies which observed alterations in trophic factors secreted by collapsed myofibers affecting MuSC proliferation^39^ and niche-secreted factors like WISP1 secreted by FAPs or Oncostatin M secreted by myofibers, both affecting MuSC behavior^3,40^. After denervation, the number of MuSCs and especially proliferating MuSCs increased, which is in line with a previous report demonstrating that MuSCs sense the loss of NMJ integrity after denervation and adapt in order to support re-innervation^17^. Surprisingly, a drastic decline of Pax7^+^ MuSCs was reported in immobilized fetal chicken^41^. Muscle contractions were experimentally blocked leading to atrophy and reduced numbers of myogenic progenitors. In DEN muscles, contractions are also disabled but no decline in MuSC numbers was observed. Possibly, the absence of the nerve in DEN muscles is inducing regeneration-like signals causing MuSCs to break quiescence and activate while the contact with the nerve is still established in the immobilization model. On the contrary, recent studies by the Puri group^9,10,42^ did not observe major changes in the MuSC population, but rather in muscle-resident glia cells, activated fibroblasts and FAPs after loss of innervation. By performing extensive scRNAseq and snATAC-seq analyses, Nicoletti et al.^42^ identified muscle resident glia cells and Thy1/CD90-expressing mesenchymal cells as the two main cell types responding to denervation. Moreover, both cell types communicated via NGF/ NGFR signaling in order to repair the damaged NMJ, which is hampered by the denervated environment. Earlier, Proietti et al. identified a muscle-resident Itga7^+^ glia cell population that shows substantial alterations in the gene expression landscape after loss of innervation accompanied by an increase in cell number^10^. In our study, MuSCs were identified as α7-integrin^+^ - Sca-1^-^ - CD11b^-^ - CD31^-^ - CD45^-^ and therefore, this population could also contain muscle resident Itga7^+^ glia cells. Although there was no difference observed in the percentage of Pax7^+^ cells within the α7-integrin^+^ cell population between Sham and DEN muscles, only 85% of the cultured α7-integrin^+^ cells were Pax7^+^ . Thus, we cannot exclude that other α7-integrin expressing cell types were co-isolated with the MuSC population. To further determine whether this is the case, we used the transcriptome data set of Sham and DEN MuSCs and investigated the expression of glia cell and MuSC markers suggested by Proietti et al. Interestingly, some of the glia cell markers, like Ngfr, were indeed significantly enriched in our FACS-isolated Itga7^+^ cells (data not shown) while others showed a reduction (e.g. Mbp). Therefore, it is possible that MuSCs either changed their expression profile towards a glia cell like gene signature following denervation or that the FACS-isolated Itga7^+^ cells also contain other cells, like muscle-resident glia cells. Although we cannot rule out the possibility that our MuSC data sets contain small fractions of other cell populations, we did observe a clear and reproducible increase in the number of Pax7^+^ cells on muscle cross sections from denervated mice, suggesting that muscle stem cells display significant alterations following denervation. Interestingly, the higher number of myogenic cells per myofiber after denervation observed here was sustained over the time of culture while the activation potential was not altered, suggesting that MuSCs are still functional after denervation when removed from the denervated microenvironment. This further supports our notion that factors from the niche – including changes in the secretome of myofibers – are the main cause of changes in MuSCs after denervation and that alterations in MuSCs are at least partially reversible. Indeed, we found that MuSC engraftment and fusion potential were not affected by host or donor innervation state. This is in line with a study showing that transplantation of MuSCs from an innervated or denervated donor into long-term denervated muscle produced comparable numbers of donor MuSC-derived myofibers^12^, indicating that the intrinsic fusion potential of MuSCs is independent of innervation. In contrast, a recent study observed an increase in donor MuSC-derived myofibers after transplantation when muscle injury was combined with a nerve crush injury in the recipient mice 28 days after transplantation^43^. Possibly, the reduced severity of the nerve crush injury created an environment that allowed for more efficient MuSC engraftment or differentiation dynamics.

The changes in the niche – including alterations in the myofiber secretome – also affect MuSC behavior after injury and thereby regeneration of skeletal muscle. The effects on MuSCs observed in this study are immediate, since denervation in combination with a CTX injury resulted in impaired regeneration, suggesting that a therapy for improving MuSC functionality should start immediately after such an injury. While a study in long-term denervated muscles (3–12 months) did not observe a reduction in the regenerative potential of the resident MuSCs^12^, the acute denervation performed here at the same time as the muscle injury seemed to impact MuSCs differently compared to a muscle injury in long-term denervated muscle. A recent study showed that the combination of muscle injury with a nerve crush led to an increase of eMHC positive myofibers^43^, which is similar to the results obtained here after sciatic nerve transection. Furthermore, it was shown that reinnervation after injury of skeletal muscle is taking place around day 8 after injury^21^. This is suggesting that the immediate loss of innervation combined with the prevention of reinnervation is causing the impaired regeneration of skeletal muscle observed here.

In conclusion, we show that denervation leads to changes in the MuSC niche, especially the myofiber secretome including Tgfb1, which then causes alterations in MuSCs, such as enhanced *Junb* expression and impaired functionality. The findings presented here unravel some of the components and pathways that are causing impaired MuSC functionality after denervation and could therefore help to prevent or revert this phenotype, thereby improving regeneration of skeletal muscle in neuromuscular pathologies and after acute injuries.

## Methods

### Animals and surgical procedures

All animal procedures were performed in accordance with the national regulations for animal experimentation and approved by the Thüringer Landesamt für Verbraucherschutz (license number FLI-17-015).

Young (2–6 month) male C57BL/6 J mice were obtained from Janvier or bred in-house. CAG-GFP (JAX: 003291)^23^ donor mice were bred in-house. All mice were housed at the Leibniz Institute on Aging – Fritz Lipmann Institute under specific-pathogen-free conditions on a 12/12 h light/dark cycle in individually ventilated cages under constant humidity and temperature and received standard chow and water *ad libitum* according to the directive 2010/63 EU and GV SOLAS. Mice were sacrificed using CO_2_ inhalation at the time points indicated in the respective schematics in the figures.

The *tibialis anterior* (TA) muscle was injected with 50 µl of 20 µM cardiotoxin (CTX, Latoxan) in 0.9% NaCl using a 29-gauge needle as described before^44^.

For unilateral sciatic nerve transection (denervation, DEN), a small skin incision was made at the mid-thigh region, the sciatic nerve was exposed and a ca. 0.3 cm long piece of nerve was cut out. In the control animals (Sham surgery), the sciatic nerve was located without transection.

Young male and female donor CAG-GFP mice were subjected to Sham or DEN surgery, sacrificed 21 days later and 10.000 FACS-isolated GFP positive MuSCs in 25 µl 0.9% NaCl were immediately injected with a 29 gauge needle intramuscularly into the TA muscle of C57BL/6 J recipient mice. Recipient mice received a CTX injury of the TA muscle together with either Sham or DEN surgery two days prior to transplantation and an implantation of an osmotic pump containing 25 mg/ml FK-506 in 70% ethanol with a release rate of 0.25 µl/h over a duration of 28 days.

For all surgical procedures (performed under anesthesia), analgesics (1 mg/ kg Meloxicam subcutaneously) were administered.

### Muscle cross section analysis

Muscles were harvested and used for cryosectioning and immunofluorescence staining as described earlier^44^. TA muscles transplanted with GFP positive MuSCs were fixed in 2% PFA for 3 h at room temperature (RT) and a sucrose gradient was applied for three consecutive nights (10% → 20% → 30% sucrose in ddH_2_O) before freezing in liquid nitrogen. Sections were not additionally fixed for eMHC staining. Cross sections from transplanted muscles were additionally fixed in 2% PFA for 30 min before immunofluorescence staining. Afterwards, antigen retrieval was performed in a decloaking chamber (Biocare Medical) for 10 min at 95 °C using 1 x HIER antigen retrieval buffer (Abcam) before the staining was proceeded. Primary and secondary antibodies are listed in Table 1 and 2 in the supplemental information. Microscopic images were acquired on an upright Axio imager microscope from Zeiss with a 20x objective.

Masson Trichrome staining was performed with the HT15-1KT kit (Sigma Aldrich) according to the manufacturer’s protocol and sections were analyzed using Qupath 0.2.3^45^.

### Single myofiber analysis

Single myofiber isolation was performed as described earlier^46^. Briefly, *extensor digitorum longus* (EDL) muscles were carefully excised from tendon to tendon and digested in a 0.2% collagenase (w/v) solution for 60–90 min at 37 °C, thereby eliminating cells that are not under the basal lamina. Single myofibers were obtained by carefully triturating the digested muscle with a horse serum coated glass Pasteur pipet with a broken-off heat polished tip. 50–100 single, non-contracted myofibers were used for each experimental condition. For RNA sequencing, myofibers were directly transferred to TRIzol reagent for RNA isolation. For immunofluorescence staining, myofibers were either immediately fixed after isolation or cultured for up to 72 h at 37 °C and 5% CO_2_ before fixation and staining. Recombinant proteins or solvent controls were directly added to the medium and myofibers were incubated for the respective amount of time. For Opn, Ostn and Chrd recombinant proteins 0.1% BSA in PBS was used as a solvent control, for Tgfb1 0.1% BSA in 10 mM citric acid was used. Opn alone was used in a concentration of 3 µg/ml. In the recombinant protein cocktail, all factors were used in a final concentration of 1 µg/ml. Tgfb1 recombinant protein was used in a final concentration of 20 ng/ml.

Myofiber supernatants were collected from cultured myofibers at the respective times and immediately used in a 1:1 ratio with fresh culture medium. Control (msIgG) or blocking antibody (1 µg/ml nTgfb1) were directly added to the medium and myofibers were incubated for the 72 h. The components used for myofiber experiments are listed in Table 3 in the supplemental information.

### ELISA

Myofiber culture supernatants were collected after 4 h for ELISA analysis of Opn and Tgfb1 protein according to the manufacturer’s protocols (Thermo Fisher Scientific, EMSPP1, Invitrogen™ BMS6084). The absorbance was measured at a wavelength of 450 nm and concentrations of the candidate proteins were determined from the standard curve using an ELISA calculator (https://www.arigobio.com/ELISA-calculator).

### FACS-isolation of MuSCs

Hind limb muscles were harvested, minced with scissors and digested enzymatically with collagenase B (10 mg/ml, Roche) and dispase II (4 mg/ml, Roche) in PBS at 37 °C for 30 min under trituration every 10 min. The suspension was mixed with growth medium (Ham’s F-10 Nutrient Mix + 20% FBS + 1x P/S + 2.5 ng/ml bFGF), filtered through a 74 µm pore size strainer (Corning Life Sciences), spun down and resuspended in FACS buffer (PBS with 2% FBS) with the respective antibodies listed in Table 4 in the supplemental information. After 15 min incubation on ice, samples were spun down, resuspended in FACS buffer and filtered through a 35 µm nylon mesh (Corning Life Sciences) before 1 mM SYTOX^TM^ Blue dead cell stain was added 1:1000. MuSCs were identified as α7-integrin^+^ - Sca-1^-^ - CD11b^-^ - CD31^-^ - CD45^-^ - Sytox^-^ using a FACSAria III with Diva software v8.0.1 (BD Life Sciences). For MuSCs from CAG-GFP mice cells were additionally sorted for GFP signal. Isolated α7-integrin^+^ cells from muscles of Sham or DEN operated mice were seeded on a collagen – coated cell culture plate and allowed to attach in growth medium at 37 °C, 5% CO_2_ and 20% O_2_. Afterwards, cells were stained for the canonical MuSC marker Pax7 and the percentage of Pax7^+^ cells was determined.

### Myoblast culture

FACS-isolated MuSCs were seeded on collagen-coated culture plates in growth medium (see “FACS-isolation”) at 37 °C, 5% CO_2_ and 20% O_2_. For analysis of *Junb* gene expression and protein abundance, myoblasts were seeded and allowed to attach for at least 24 h. Then, medium was replaced by medium containing the solvent control (0.1% BSA in 10 mM citric acid), 20 ng/ml Tgfb1 recombinant protein or Sham/ DEN myofiber supernatant. At the respective time point, cells were harvested for RNA or protein isolation.

### RNA sequencing and analysis

Sequencing of RNA samples was performed using Illumina’s next-generation sequencing methodology^47^. TA muscles or EDL myofibers were homogenized in TRIzol reagent and RNA was isolated according to the manufacturer’s protocol (VWR, 30-2010). RNA was resolved in nuclease free water and RNA quality was assessed with the Agilent 4200 Tapestation and RNA screen tape. Library preparation was performed using the NEBNext Ultra II directional RNA kit with polyA molecule selection and unique dual indexing. For sequencing, the NovaSeq6000 SP system was used (single-end, 101 bp).

MuSCs were FACS-isolated and sorted directly into lysis buffer containing β-mercaptoethanol from the RNeasy Micro Kit (Qiagen) and samples were processed according to the manufacturer’s protocol. RNA quality was assessed with the Agilent 2100 Bioanalyzer and RNA 6000 pico kit. Library preparation was performed with a two-step approach: (1) introducing 4 ng of total RNA to the SMART-Seq® v4 Ultra® Low Input RNA Kit for Sequencing (Takara) for full-length cDNA synthesis was followed by (2) introducing 300 pg of full-length cDNA to the Nextera XT DNA Library Preparation Kit (Illumina). For sequencing, the NextSeq 500 high-output system was used (single-end, 75 bp).

Data were extracted using the bcl2FastQ v2.20.0.422 conversion software. Adapter trimming was performed with CutAdapt v2.1 (-m 30 –a ADAPER-SEQUENCE)^48^. Mapping of data was done using TopHat v2.1 (–no-convert-bam –no-coverage-search -x 1 -g 1)^49^ with the *Mus musculus* GRCm38.100 genome as a reference^50^. Reads per gene were counted with featureCounts v1.6.5 (-s 2: TA/EDL or –s 0: MuSCs)^51^. The counts were imported into the statistical computing environment R (v.3.6.3). DEseq2 package v1.26.0^52^ was applied in order to detect differentially expressed genes (DEGs). The *p*-values were calculated using the Wald significance test followed by correction for multiples testing using the Benjamini & Hochberg procedure. Genes with adjusted *p*-values < 0.05 were considered to be differentially expressed. Fold changes (and log2 fold changes) were calculated based on reads per million mappable reads (RPMs).

The multidimensional scaling (MDS) was done as follows: Based on gene counts a matrix of spearman correlation coefficients of all vs. all samples was made (R function cor: method = ”spearman”). For computing the Euclidean distances, the correlation coefficients were subtracted from 1 (1 rs) followed by computing of distances (R function dist: method = “euclidean”). The distance matrix was used to perform the MDS (R function cmdscale: eig = TRUE, k = NumberOfSamples-1). First vs. second dimension data were plotted using R package ggplot2 v3.3.2^53^. By manual inspection of the MDS plot, sample “MuSC 09” was determined to be an outlier and was excluded from further analysis (plot not shown).

The DAVID database (https://david.ncifcrf.gov/tools.jsp) was used to screen for predicted secreted/ extracellular factors. Venn diagrams were generated using the online tool Interactivenn^54^ and edited using Adobe Illustrator.

Gene set enrichment analysis (GSEA) was done with the gseGO or gseKEGG functions of the R-package clusterProfiler v4.2.2^55^ based on sorted log2 fold change values. For this, all genes from the DESeq2 analysis were considered, having a valid log2 fold change value. Gene ontology (GO) terms were consolidated with the simplify function. Prior to gseKEGG, the gene identifiers were mapped from Ensembl to Entrez scheme. Figures were generated with the dotplot function. DEGs of MuSCs after denervation were compared to two lists of transcription factors (TFs), downloaded from https://github.com/gifford-lab/ReprogrammingRecovery/blob/main/data/mouse_ensemble_tfs_from_lambertetal_isyes.unique.txt^25^ (*n* = 1374) and http://bioinfo.life.hust.edu.cn/static/AnimalTFDB3/download/Mus_musculus_TF^26^ (*n* = 1636). Both lists were intersected with all detected genes in MuSCs after denervation to exclude TFs that are not represented in our analysis resulting in 1164 and 1325 TFs, respectively, which were compared to 2613 DEGs and visualized as a Venn diagram. To analyze pathways that are changed in MuSCs upon denervation, we performed overrepresentation analysis (ORA) with WebGestaltR (0.4.6). Here, genes were included in the analysis with a log2 fold change >0.5 and a padj < 0.05. To perform a differential analysis between Junb and non-Junb targets, the same analysis was performed similarly. The enrichment scores were then used to identify changes that are specific for Junb targets: Enrichment_ratio (Junb) / Enrichment_ratio (non-Junb). To compare the transcriptional changes occurring in our DEN data sets to the changes that occur during regeneration upon muscle injury, we downloaded the gene expression data from the following data set from GEO: GSE121589^56^. Here, genes that were differentially expressed upon denervation, were tested in the regeneration data sets using Z-score normalized gene expression data of the published data set and plotted as a heatmap in R by ComplexHeatmap (2.15.4).

### Proteomic analyses

MuSCs were FACS-sorted into 2x lysis buffer and processed for MS-based proteome analysis as described^57^. A minimal cell number of ~100.000 cells per sample was used for each replicate. Briefly, samples were lysed and homogenized, boiled at 95 °C for 10 min and sonicated at 20 °C. After incubation with dithiothreitol (final concentration 10 mM, 45 °C) and addition of iodoacetamide (final concentration 15 mM, RT in the dark), samples were incubated with eight volumes ice-cold 100% acetone at -20 °C over night before washing twice with 80% ice-cold acetone. The pellet was air-dried, digested (3 M Urea and 100 mM HEPES, pH = 8) and sonicated. Addition of Lys-C (final concentration 0.05 µg/µl, 4 h at 37 °C) was followed by 1:1 dilution with HPLC water and Trypsin digestion (1:100 enzyme: protein ratio) over night at 37 °C. Samples were acidified by addition of 10% trifluoroacetic acid to a final of 1% and desalted using a Waters Oasis HLB μElution Plate 30 µm (Waters, #186001828BA) according to the manufacturer’s instruction. Eluted samples were dried in a SpeedVac at 45 °C before reconstitution to 1 µg/µl in 10 µl of buffer (0.1% formic acid in 5% acetonitrile in HPLC water) and homogenization. The iRT kit was used as an internal control in a dilution recommended by the manufacturer. Peptides were separated using the nanoAcquity UPLC MClass system (Waters) with the Proxeon nanospray source. For data acquisition and processing of the raw data Xcalibur v4.0, Tune v2.1 (Thermo Fisher) and Spectronaut v13.1 (Biognosys) were used with the default settings.

### cDNA synthesis and qRT-PCR

cDNA synthesis was performed with the iScriptTM cDNA synthesis kit (Bio-Rad) according to the manufacturer’s protocol. Quantitative real time PCR (qRT-PCR) was performed using diluted cDNAs with the iQ SYBR green supermix (Bio-Rad) according to the manufacturer’s protocol on a Stratagene 3000 cycler (Agilent Technologies) with the MX pro software. Expression levels are presented relative to a housekeeping gene and were calculated from the C_T_-values according to the 2^-ΔΔCt^ method^58^. Each cDNA template was used in technical duplicates or triplicates; mean values were used for further calculations. Primers are listed in Table 5 in the supplemental information.

### Western blot

Snap frozen muscle, isolated myofibers or myoblasts were lysed and homogenized in RIPA buffer containing PhosSTOP (Roche) and Protease STOP (Roche) for 20 min on ice. Samples were boiled at 99 °C for 5 min and 20–30 µg of protein were used for separation on Bis-TRIS gels by SDS-PAGE and blotting onto a PVDF membrane. Membranes were stained with Ponceau S solution as loading control. Membranes were incubated in 5% milk in TBS-T for 1 h at RT followed by incubation with the respective primary and secondary antibodies in 5% milk in TBS-T. Detection was carried out using the Pierce™ ECL Western Blotting Substrate (Thermo Fisher Scientific) and the ChemiDoc MP imaging system (Bio-Rad).

Primary and secondary antibodies are listed in Table 1 and 2 in the supplemental information.

### Statistical analyses

All figures were assembled with Adobe Illustrator. Statistical analyses were performed using GraphPad Prism. Each experiment was performed at least in biological triplicates. Data are represented as mean and error bars represent standard deviation (SD). The statistical test applied for each experiment is specified in the respective figure legend. The statistical significance is indicated in the figures by stars, with a *p*-value lower than 0.05 being considered significant. A *p*-value lower than 0.05 is symbolized by one star (*), *p* < 0.01 by two stars (**), *p* < 0.001 by three stars (***) and *p* < 0.0001 by four stars (****).

### Reporting summary

Further information on research design is available in the Nature Research Reporting Summary linked to this article.

### Supplementary information

Supplemental Material

Reporting Summary

## Data Availability

The RNA sequencing data discussed in this publication have been deposited in NCBI’s Gene Expression Omnibus^59^ and are accessible through GEO Series accession numbers GSE217928 and GSE217929. Sample IDs are listed in Table 6 in the supplemental information. The mass spectrometry proteomics data have been deposited to the ProteomeXchange Consortium via the PRIDE^60–62^ partner repository with the dataset identifier PXD036993.
