# Supplementary material for: Denervation alters the secretome of myofibers and thereby affects muscle stem cell lineage progression and functionality
Source: NPJ Regen Med. 2024 Mar 1;9:10. doi: 10.1038/s41536-024-00353-3 (PMC10904387; doi:10.1038/s41536-024-00353-3)
Supplement: Supplementary file 1 — Supplemental Material [file 41536_2024_353_MOESM1_ESM.pdf]

Denervation alters the secretome of myofibers and thereby affects muscle stem cell lineage progression and functionality

Henriette Henze[1], Sören S. Hüttner[1], Philipp Koch[1], Svenja C. Schüler[1], Marco Groth[1], Björn von Eyss[1] and Julia von Maltzahn[1,2]

Supporting information

Supporting figures:

Supplemantary Figure 1

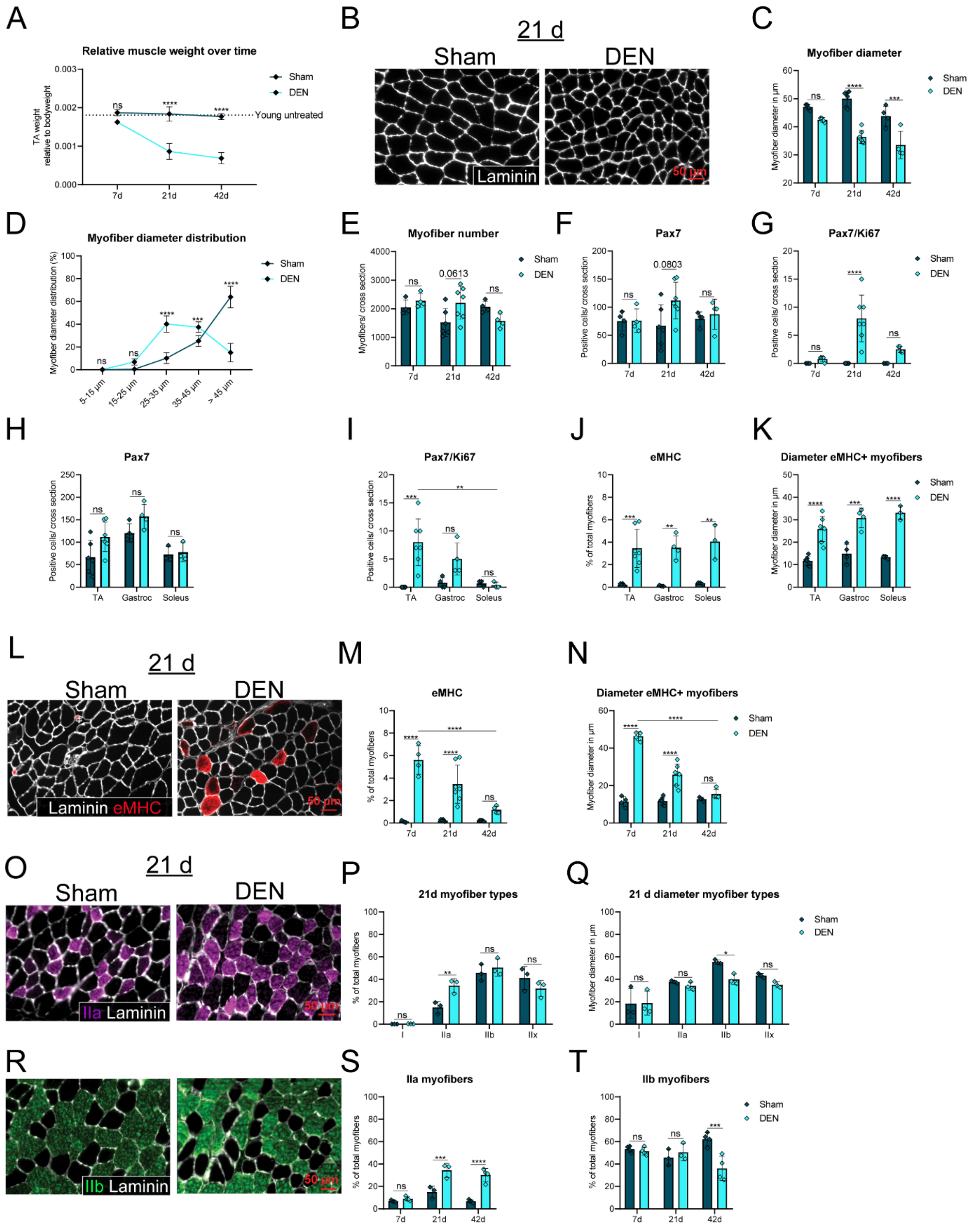

Supplementary Figure 1: Functional motor innervation is important for MuSC homeostasis and myofiber type specification. A) TA muscle weight relative to bodyweight at different time points after surgery. The dotted line represents the average TA muscle weight relative to body weight of young (2 – 6 months) untreated male C57BL/6J mice. n= 16 for young untreated mice and n= 4 – 7 for Sham and DEN mice. **B)** Immunofluorescent staining of TA muscle cross sections for Laminin 21 d after surgery. Scale bar is 50 µm. **C – E)** Quantification of Laminin staining for all time points. Myofiber diameter (C), myofiber diameter distribution (D) and myofiber number (E) are shown. **F, G)** Quantification of Pax7 positive cells per section (F) and Pax7/Ki67 double positive cells per section (G) at different time points after surgery. **H – K)** Quantification of different stainings at 21 d after surgery of TA, Gastrocnemius or Soleus muscle. Shown are Pax7 positive cells per section (H), Pax7/Ki67 double positive cells per section (I), percentage of eMHC positive myofibers (J) and diameter of eMHC positive myofibers (K). **L)** Immunofluorescent staining of TA muscle cross sections for eMHC (red) and Laminin (white) 21 d after surgery. Scale bar is 50 µm. **M, N)** Quantification of eMHC staining for all time points. Percentage of eMHC positive myofibers of total myofibers (M) and myofiber diameter of eMHC positive myofibers (N) are shown. **O, R)** Immunofluorescent staining of TA muscle cross sections for Laminin (white) and type IIa myofibers (purple) or type IIb myofibers (green) 21 d after surgery. Scale bar is 50 µm. **P, Q, S, T)** Quantification of myofiber type characteristics at different time points after surgery. **P)** Myofiber type composition in percent of total myofiber number at 21 d after surgery. **Q)** Myofiber diameter at 21 d after surgery for all myofiber types. **S, T)** Percentage of type IIa (S) and IIb (T) myofibers of total myofibers at different time points after surgery. n= 3 – 7 animals per surgery group, each data point represents one animal. Statistical testing was done by Two-way-ANOVA with post-hoc Tukey`s multiple comparisons test. Error bars represent SD. ns= not significant, (*) $p < 0.05$, (**) $p < 0.01$, (***) $p < 0.001$, (****) $p < 0.0001$.

Supplemantary Figure 2

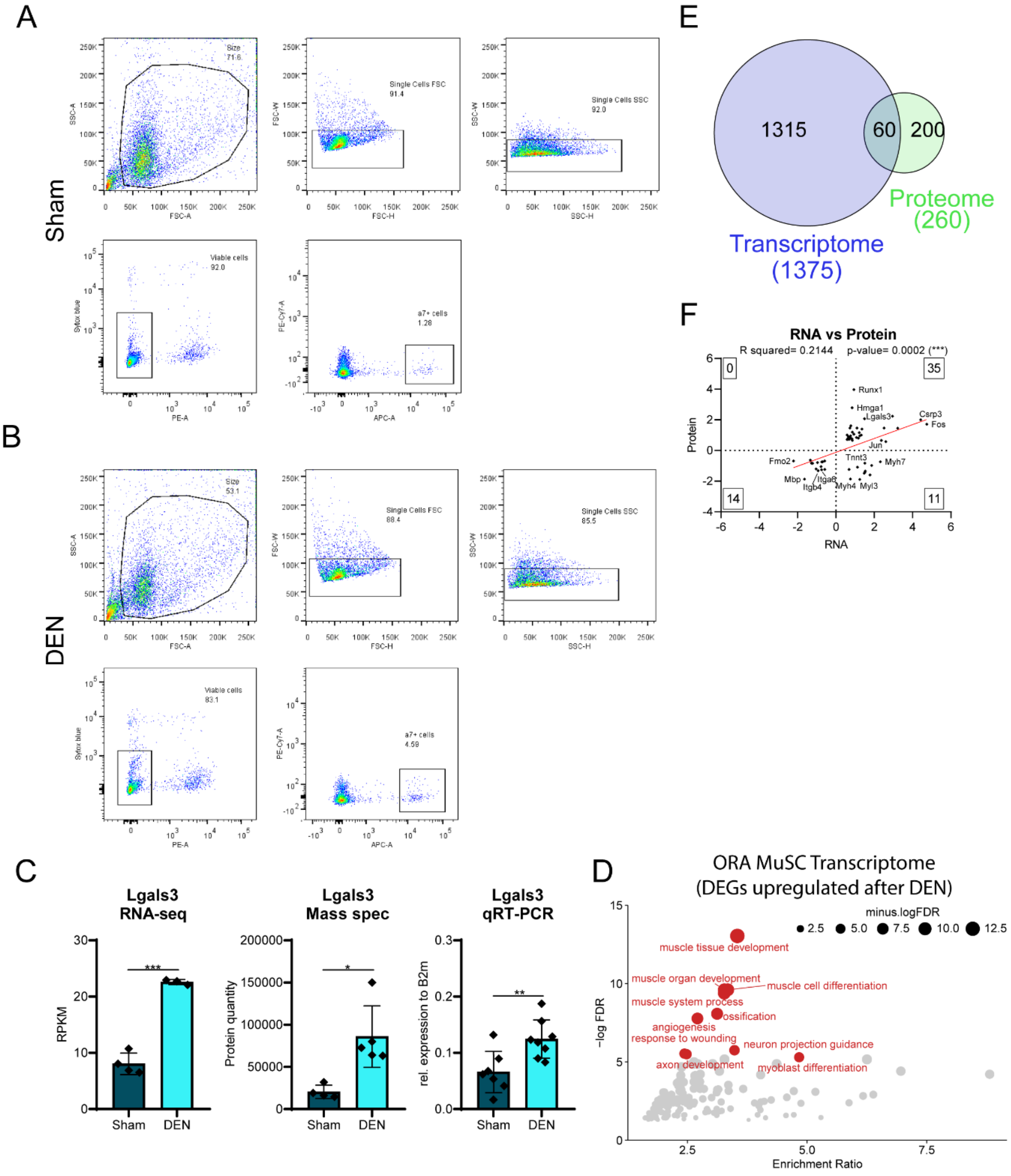

Supplementary Figure 2: MuSC Omics analysis. Male C57BL/6J mice were subjected to either Sham or DEN surgery. At 21 days after surgery, hind limb muscles were harvested for FACS-isolation of MuSCs. **A, B)** Representative FACS plot of hind limb muscles from a Sham (A) or

DEN (B) mouse. MuSCs were identified as α7-integrin+ - Sca-1$^-$ - CD11b$^-$ - CD31$^-$ - CD45$^-$ - Sytox$^-$. **C)** Validation of Omics data (left) by qRT-PCR for Lgals3 mRNA (right). n= 3-4 (RNA-seq) and 7 – 8 (qRT-PCR), each data point represents one animal. Statistical testing was done by unpaired two-tailed t-test with Welch`s correction. Error bars represent SD. (**) $p < 0.01$. **D)** Overrepresentation analysis (ORA) of MuSC transcriptome after DEN. Genes significantly upregulated after DEN (adj. p – value <0.05, log2fc > 0.5) are involved in the depicted processes. The top 10 overrepresented processes are highlighted in red. **E)** Overlap analysis of DEGs (blue) and DAPs (green) (adj. p – value <0.05, log2fc </> - 0.58/+0.58) in MuCSs after DEN. **F)** Correlation of 60 shared factors between MuSC transcriptome and proteome (log2 fold changes are plotted). Statistical testing was done by Pearson correlation analysis. (***) $p < 0.001$.

Supplementary Figure 3

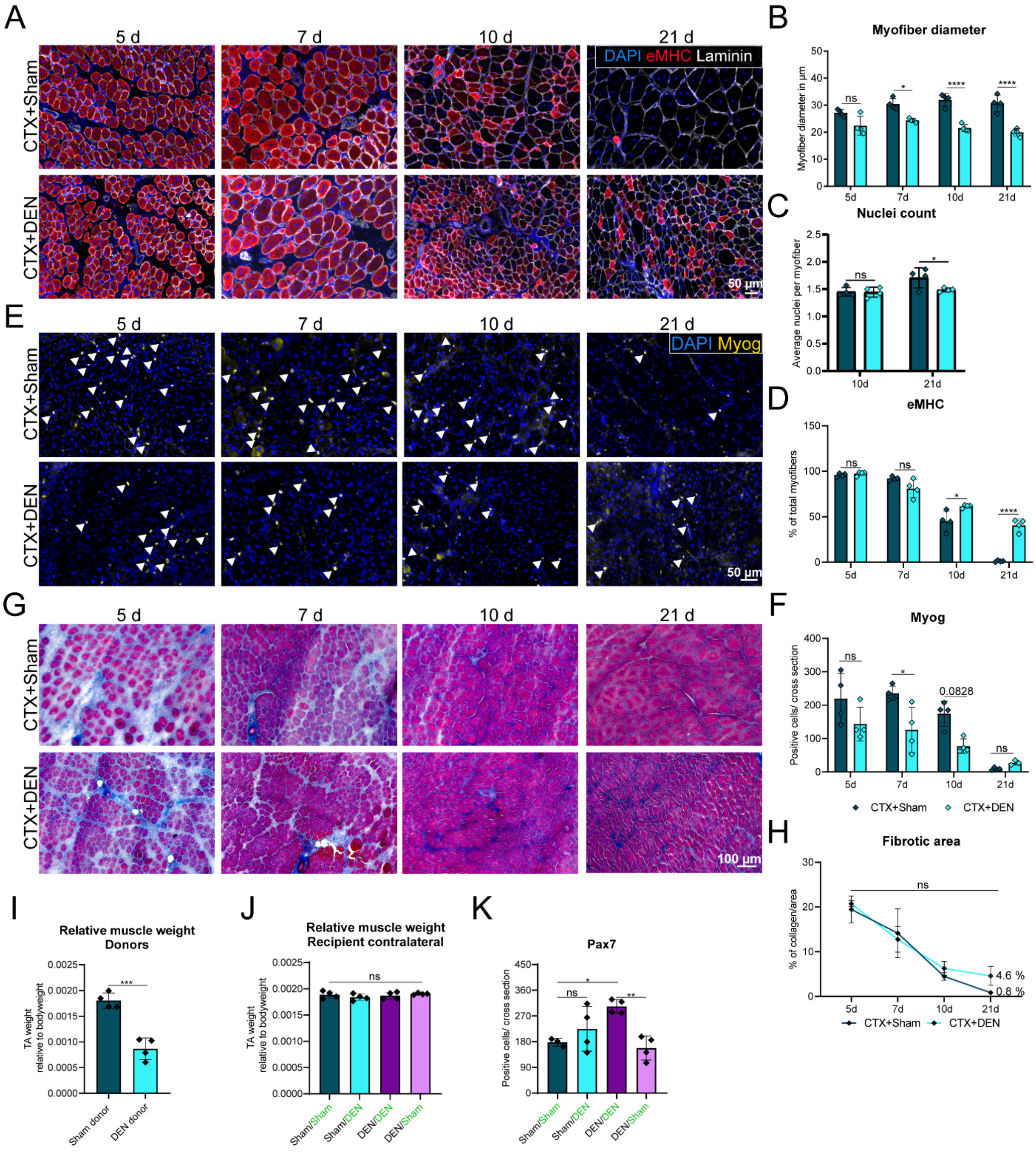

Supplementary Figure 3: Myofiber niche alterations are driving MuSC dysfunction in denervated skeletal muscle. A) Immunofluorescent staining of TA muscle cross sections of CTX+Sham and CTX+DEN operated mice at different time points after surgery. Myofibers are stained with Laminin (white), newly formed myofibers are stained with eMHC (red) and nuclei are counterstained with DAPI (blue). Scale bar is 50 µm. B – D) Quantification of (A). E) Immunofluorescent staining of TA muscle cross sections of CTX+Sham and CTX+DEN operated mice at different time points after surgery. White arrowheads indicate Myog positive cells (yellow) and nuclei are counterstained with DAPI (blue). Scale bar is 50 µm. F) Quantification of (E). G) Masson`s Trichrome staining of TA muscle cross sections at respective time points after surgery. Blue stained areas indicate collagenous/ fibrotic tissue while myofibers are stained pink. Scale bar is 100 µm. H) Quantification of (G). n= 4 animals per surgery group at each time point. Statistical testing was done by Two-way-ANOVA with post-hoc Tukey`s multiple comparisons test. Error bars represent SD. ns= not significant, (*) $p < 0.05$, (****) $p < 0.0001$. I) TA muscle weight relative to bodyweight for Sham or DEN donor mice at 21 days after surgery. J) TA muscle weight of recipient contralateral leg relative to bodyweight for each group at 21 days after MuSC transplantation. K) Quantification of Pax7 positive cells per cross section. Donor indicated in green. n= 3 – 4 animals per surgery group. Statistical testing was done by unpaired two-tailed t-test with Welch`s correction (I) or One-way-ANOVA with post-hoc Tukey`s multiple comparisons test (J and K). Error bars represent SD. ns= not significant, (*) $p < 0.05$, (**) $p < 0.01$, (***) $p < 0.001$.

Supplementary Figure 4

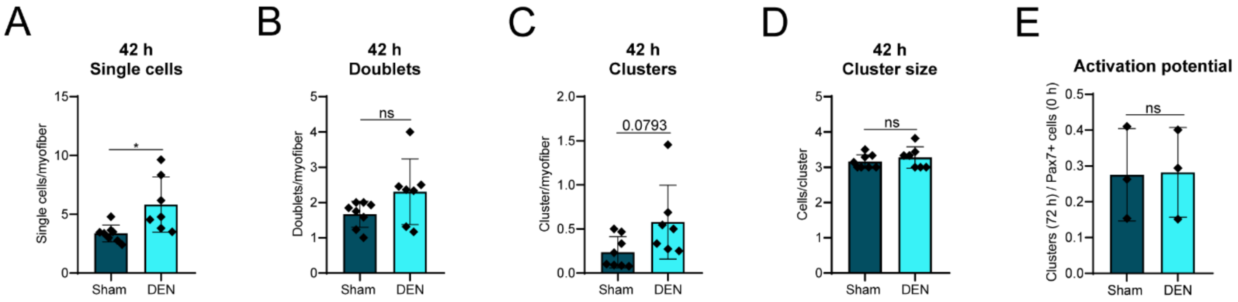

Supplementary Figure 4: The immediate MuSC niche is disrupted after denervation. A, B) Quantification of single cells (A) and doublets (B) per myofiber after 42 h of culture. **C, D)** Cluster number per myofiber (C) and average cluster size (D). **E)** Activation potential of MuSCs (number of clusters at 72 h divided by number of Pax7+ MuSCs at 0 h). Statistical testing was done by unpaired two-tailed t-test with Welch`s correction. n= 3 – 8 animals per surgery group. Error bars represent SD. ns= not significant, (*) $p < 0.05$.

Supplementary Figure 5

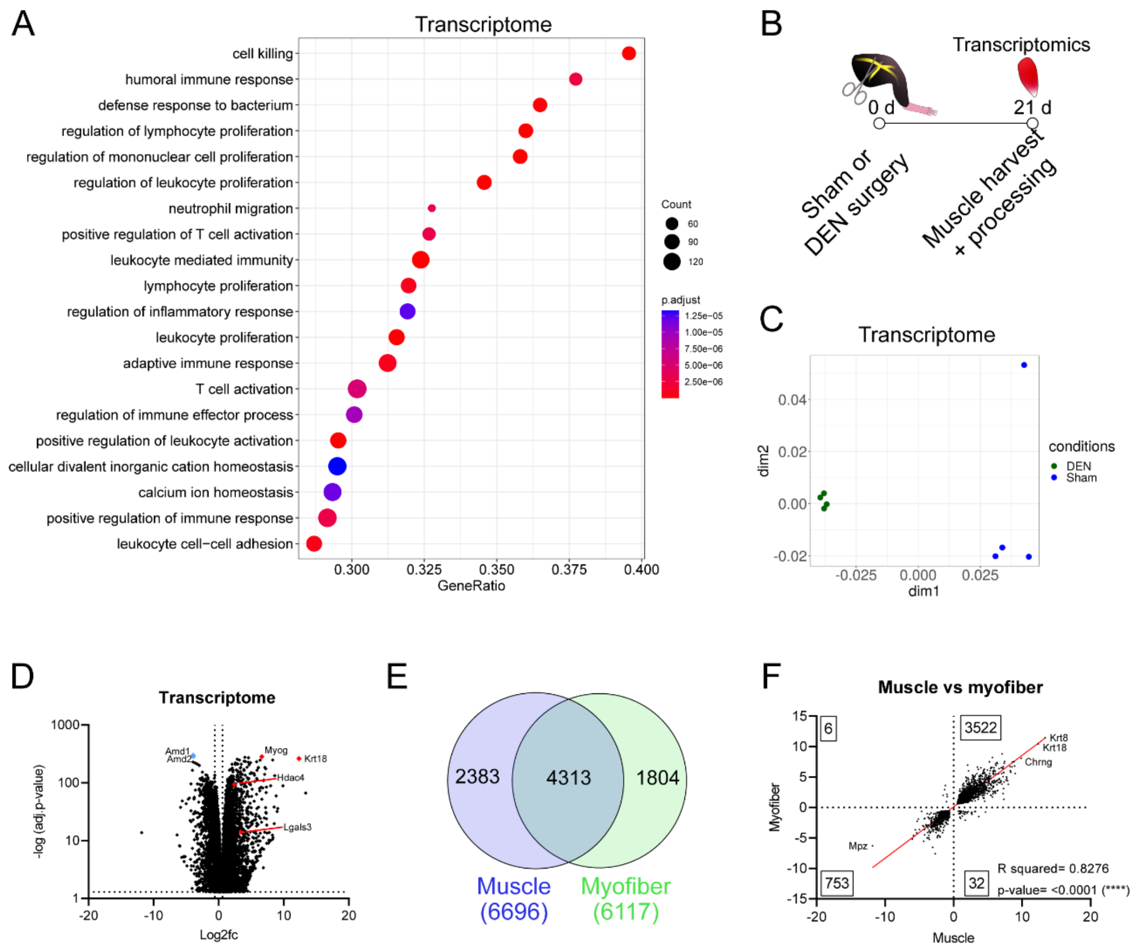

Sup. Figure 5: Whole muscle and myofiber transcriptome are massively altered after skeletal muscle denervation. A) 20 most significant biological processes in myofibes after denervation identified as activated via GSEA. GeneRatio represents the fraction of enriched genes within a GO term. **B)** Experimental scheme. Male C57BL/6J mice were subjected to either Sham or DEN surgery. At 21 days after surgery, TA muscles were harvested for transcriptome analysis. **C)** MDS plot of TA muscle transcriptome. **D)** Volcano plot of DEGs (adj. p – value <0.05) in TA muscles of DEN mice. Represented is the log2 fold change relative to expression in Sham samples, with selected downregulated genes in blue and upregulated genes in red. Dotted lines mark log2 fold change of -/+ 0.58 and adj. p –value <0.05. **E)** Venn diagram of DEGs (adj. p –

value <0.05, log2fc </> -0.58/+0.58) in whole muscle tissue (blue) vs isolated myofibers (green) after DEN. **F)** Correlation of 4313 shared genes in transcriptomes of whole muscle and isolated myofibers (log2 fold changes are plotted). Statistical testing was done by Pearson correlation analysis. (****) $p < 0.0001$.

Supplementary Figure 6

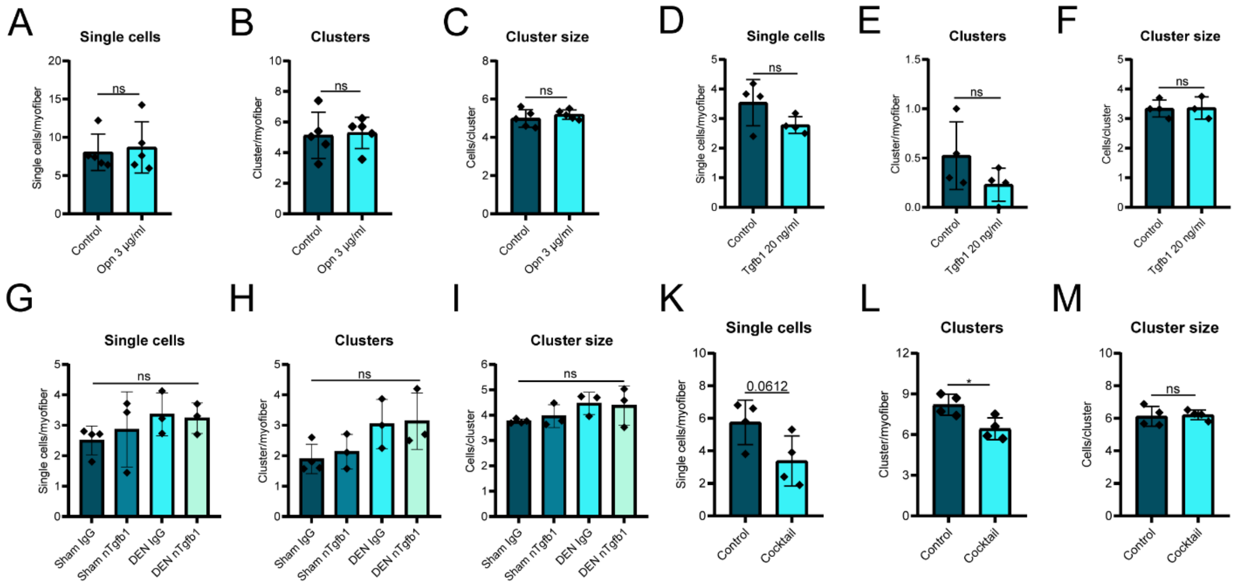

Supplementary Figure 6: Effects of denervation-induced alterations in the myofiber secretome on MuSCs . A – C) Quantification of single cells (A), clusters (B) and cluster size (C) after 72 h of culture with Opn recombinant protein (3 µg/ ml) or solvent control (0.1 % BSA in PBS). **D – F)** Quantification of single cells (D), clusters (E) and cluster size (F) after 48 h of culture with Tgfb1 recombinant protein (20 ng/ ml) or solvent control (0.1 % BSA in 10 mM citric acid). **G – H)** Quantification of single cells (G), clusters (H) and cluster size (I) after 72 h of culture with Sham or DEN SUP together with either normal msIgG or nTgfb1 (1 µg/ ml) blocking antibody. **(K – M)** Quantification of single cells (K), clusters (L) and cluster size (M) after 72 h of culture with recombinant protein cocktail (Opn, Ostn, Chrd, 1 µg/ ml each) or solvent control (0.1 % BSA in PBS). Statistical testing was done by unpaired two-tailed t-test with Welch`s correction (A – F and K – M) or One-way-ANOVA with post-hoc Tukey`s multiple comparisons test (G – I). n= 3 – 5 animals per surgery group. Error bars represent SD. ns= not significant, (*) $p < 0.05$.

Supplementary Figure 7

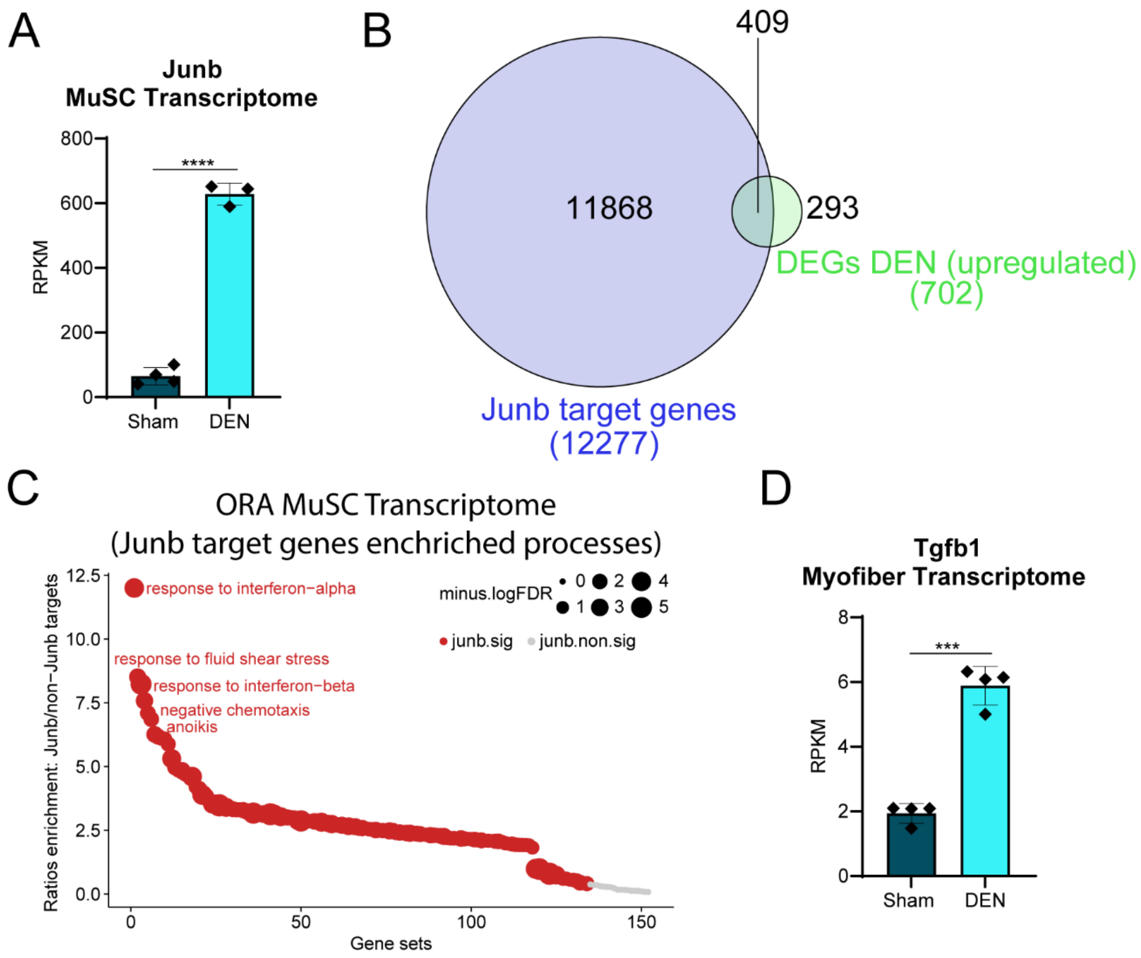

Supplementary Figure 7: Denervation induces expression of *Junb* in MuSCs. A) RPKM values for Junb transcripts in MuSCs 21 d after Sham or DEN surgery. **B)** Overlap analysis of Junb target genes (blue) and DEGs upregulated (green) (adj. p – value <0.05, log2fc > 0.5) in MuCSs after DEN. The list of Junb target genes is publicly available on TFlink.com [1]. **C)** Overrepresentation analysis (ORA) of MuSC transcriptome after DEN. Depicted are the enrichment ratios of Junb target genes over non-Junb target genes. **D)** RPKM values for Tgfb1 transcripts in myofibers 21 d after Sham or DEN surgery. n= 3 – 4 animals per surgery group. Statistical testing (A and D) was done by unpaired two-tailed t-test with Welch`s correction. Error bars represent SD. (***) p < 0.001, (****) p < 0.0001.

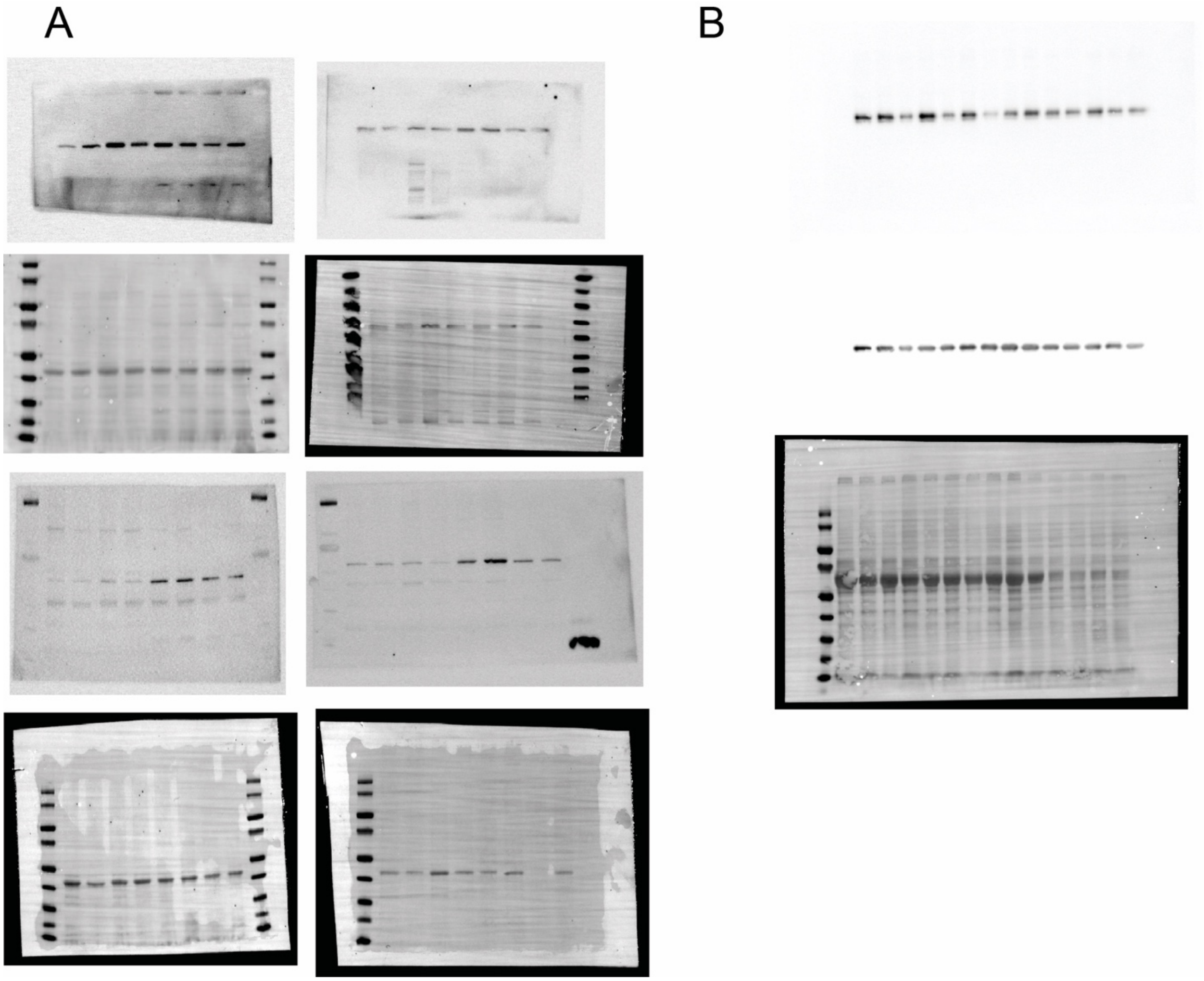

Supplementary Figure 8: uncropped original images as used in the figures. A) Immunoblots from Figure 4B. B) Immunoblots from Figure 5E**.**

Supporting tables

***Supplementary Table 1**: List of primary antibodies.*

Antibody	Species	Application/ dilution	Company, order number
**Anti-eMHC**	Mouse-IgG1	IF (undiluted)	DSHB, F1.652
**Anti-Gapdh**	Mouse-IgG	WB (1:1000)	Santa Cruz, sc-365062
**Anti-GFP**	Chicken-IgY	IF (1:500)	Abcam, ab13970
**Anti-Junb**	Rabbit-IgG	IF (1:200), WB (1:200)	Abcam, ab128878
**Anti-Ki67**	Rabbit-IgG	IF (1:200)	Abcam, ab15580
**Anti-Laminin**	Rabbit-IgG	IF (1:1000)	Sigma-Aldrich, L9393
**Anti-MHC IIa**	Mouse-IgG1	IF (undiluted)	DSHB, SC-71
**Anti-MHC IIb**	Mouse-IgM	IF (undiluted)	DSHB, BF-F3
**Anti-MyoD**	Rat-IgG2aκ	IF (1:100)	Merck, MABE132
**Anti-Myog**	Mouse-IgG1	IF (undiluted)	DSHB, F5D
**Anti-Pax7**	Mouse-IgG1	IF (undiluted)	DSHB, Pax7
**Anti-Opn**	Rabbit-IgG	IF (1:200), WB (1:1000)	Thermo Fisher Scientific, PA5-34579
**Anti-Tgfb1**	Rabbit-IgG	IF (1:200), WB (1:300)	Bioss, BS-0086R

Supplementary Table 2: *List of secondary antibodies.*

Antibody/ Conjugate	Target species	Application/ dilution	Company, order number
**Alexa Fluor™ 488**	Mouse / IgG1	IF (1:1000)	Thermo Fisher Scientific, A21121
**Alexa Fluor™ 488**	Rabbit / IgG	IF (1:1000)	Thermo Fisher Scientific, A21206
**Alexa Fluor™ 488**	Mouse/ IgM	IF (1:1000)	Thermo Fisher Scientific, A21042
**Alexa Fluor™ 488**	Rat/ IgG	IF (1:1000)	Thermo Fisher Scientific, A11006
**Alexa Fluor™ 546**	Mouse / IgG1	IF (1:1000)	Thermo Fisher Scientific, A21123
**Alexa Fluor™ 546**	Rabbit / IgG	IF (1:1000)	Thermo Fisher Scientific, A10040
**Alexa Fluor™ 647**	Mouse / IgG1	IF (1:1000)	Thermo Fisher Scientific, A21240
**Alexa Fluor™ 647**	Chicken/ IgG	IF (1:1000)	Thermo Fisher Scientific, A21449
**Alexa Fluor™ 647**	Rabbit / IgG	IF (1:1000)	Thermo Fisher Scientific, A31573
**Goat anti-mouse-HRP**	Mouse	WB (1:1500)	Dako, P0447
**Goat anti-rabbit-HRP**	Rabbit	WB (1:1000)	Dako, P0448

***Supplementary Table 3**: List of components for myofiber experiments.*

Component	Species/ Source	Final concentration	Company, order number
**Chrd recombinant protein**	Mouse	1 µg/ ml	R&D systems, 758-CN-050
**IgG isotype control**	Mouse/ IgG	1 µg/ ml	Invitrogen, 10400C
**Opn recombinant protein**	Mouse	1 µg/ ml or 3 µg/ml	R&D systems, 441-OP-050
**Ostn recombinant protein**	E. coli	1 µg/ ml	R&D systems, 9700-ON-050
**Tgfb1 blocking antibody**	Mouse-IgG1	1 µg/ ml	Invitrogen, MA5-23795
**Tgfb1 recombinant protein**	HEK293 cells	20 ng/ ml	Peprotech, 100-21

***Supplementary Table 4**: List of FACS-antibodies.*

**Component**	**Species**	**Dilution**	**Company, order number**
**Anti-alpha-7-Integrin-AF647**	Rat	1:300	AbLab, R2F2, 67-0010-05
**Anti-CD11B-PE**	Rat	1:500	BD Biosciences, M1/70, 553311
**Anti-CD31-PE**	Rat	1:500	BD Biosciences, MEC 13.3, 553373
**Anti-CD45-PE**	Rat	1:500	BD Biosciences, 30-F11, 553081
**Anti-Sca1-PE**	Rat	1:500	BD Biosciences , D7, 553108
**SYTOX^{TM} Blue dead cell stain**		1:1000	Thermo Fisher, S34857

Supplementary Table 5*: List of qRT-PCR primers.*

Gene	Species	Sequence (5' – 3')
B2m	Mouse	FW: TTCTGGTGCTTGTCTCACTGA<br>REV: AGTATGTTCGGCTTCCCATTC
Junb	Mouse	FW: TCA CGA CGA CTC TTA CGC AG<br>REV: CCT TGA GAC CCC GAT AGG GA
Lgals3	Mouse	FW:AACACGAAGCAGGACAATAACTGG<br>REV: GCAGTAGGTGAGCATCGTTGAC
Spp1	Mouse	FW: ATCTCACCATTCGGATGAGTCT<br>REV: TGTAGGGACGATTGGAGTGAAA
Tgfb1	Mouse	FW: ATG TCA CGG TTA GGG GCT C<br>REV: CTC CCG TGG CTT CTA GTG C

***Supplementary Table 6**: List of samples for RNA sequencing.*

SampleID	Tissue	Condition	Library preparation	Sequencing	Data used in figure	GEO acc. No.
TA_01	Whole muscle	DEN	NEBNext Ultra II directional RNA (polyA+)	NovaSeq 6000 (SP, 101 bp, v1.5, single-end)	Suppl. Fig. 5	GSE217928
TA_02	Whole muscle	DEN	NEBNext Ultra II directional RNA (polyA+)	NovaSeq 6000 (SP, 101 bp, v1.5, single-end)	Suppl. Fig. 5	GSE217928
TA_03	Whole muscle	DEN	NEBNext Ultra II directional RNA (polyA+)	NovaSeq 6000 (SP, 101 bp, v1.5, single-end)	Suppl. Fig. 5	GSE217928
TA_04	Whole muscle	DEN	NEBNext Ultra II directional RNA (polyA+)	NovaSeq 6000 (SP, 101 bp, v1.5, single-end)	Suppl. Fig. 5	GSE217928
TA_05	Whole muscle	Sham	NEBNext Ultra II directional RNA (polyA+)	NovaSeq 6000 (SP, 101 bp, v1.5, single-end)	Suppl. Fig. 5	GSE217928
TA_06	Whole muscle	Sham	NEBNext Ultra II directional RNA (polyA+)	NovaSeq 6000 (SP, 101 bp, v1.5, single-end)	Suppl. Fig. 5	GSE217928
TA_07	Whole muscle	Sham	NEBNext Ultra II directional RNA (polyA+)	NovaSeq 6000 (SP, 101 bp, v1.5, single-end)	Suppl. Fig. 5	GSE217928
TA_08	Whole muscle	Sham	NEBNext Ultra II directional RNA (polyA+)	NovaSeq 6000 (SP, 101 bp, v1.5, single-end)	Suppl. Fig. 5	GSE217928
EDL_09	Muscle fibers	DEN	NEBNext Ultra II directional RNA (polyA+)	NovaSeq 6000 (SP, 101 bp, v1.5, single-end)	Fig. 3	GSE217928
EDL_10	Muscle fibers	DEN	NEBNext Ultra II directional RNA (polyA+)	NovaSeq 6000 (SP, 101 bp, v1.5, single-end)	Fig. 3	GSE217928
EDL_11	Muscle fibers	DEN	NEBNext Ultra II directional RNA (polyA+)	NovaSeq 6000 (SP, 101 bp, v1.5, single-end)	Fig. 3	GSE217928
EDL_12	Muscle fibers	DEN	NEBNext Ultra II directional RNA (polyA+)	NovaSeq 6000 (SP, 101 bp, v1.5, single-end)	Fig. 3	GSE217928
EDL_13	Muscle fibers	Sham	NEBNext Ultra II directional RNA (polyA+)	NovaSeq 6000 (SP, 101 bp, v1.5, single-end)	Fig. 3	GSE217928
EDL_14	Muscle fibers	Sham	NEBNext Ultra II directional RNA (polyA+)	NovaSeq 6000 (SP, 101 bp, v1.5, single-end)	Fig. 3	GSE217928
EDL_15	Muscle fibers	Sham	NEBNext Ultra II directional RNA (polyA+)	NovaSeq 6000 (SP, 101 bp, v1.5, single-end)	Fig. 3	GSE217928
EDL_16	Muscle fibers	Sham	NEBNext Ultra II directional RNA (polyA+)	NovaSeq 6000 (SP, 101 bp, v1.5, single-end)	Fig. 3	GSE217928
MuSC_09	Muscle stem cells	DEN	SMART-Seq v4 + Nextera XT	NextSeq 500 (high-output, 75 bp, v2.5, single-end)	Outlier[a]	GSE217929
MuSC_10	Muscle stem cells	DEN	SMART-Seq v4 + Nextera XT	NextSeq 500 (high-output, 75 bp, v2.5, single-end)	Fig. 1	GSE217929

MuSC_11	Muscle stem cells	DEN	SMART-Seq v4 + Nextera XT	NextSeq 500 (high-output, 75 bp, v2.5, single-end)	Fig. 1	GSE217929
MuSC_12	Muscle stem cells	DEN	SMART-Seq v4 + Nextera XT	NextSeq 500 (high-output, 75 bp, v2.5, single-end)	Fig. 1	GSE217929
MuSC_13	Muscle stem cells	Sham	SMART-Seq v4 + Nextera XT	NextSeq 500 (high-output, 75 bp, v2.5, single-end)	Fig. 1	GSE217929
MuSC_14	Muscle stem cells	Sham	SMART-Seq v4 + Nextera XT	NextSeq 500 (high-output, 75 bp, v2.5, single-end)	Fig. 1	GSE217929
MuSC_15	Muscle stem cells	Sham	SMART-Seq v4 + Nextera XT	NextSeq 500 (high-output, 75 bp, v2.5, single-end)	Fig. 1	GSE217929
MuSC_16	Muscle stem cells	Sham	SMART-Seq v4 + Nextera XT	NextSeq 500 (high-output, 75 bp, v2.5, single-end)	Fig. 1	GSE217929

a: Outlier: sample excluded from analysis based on manual inspection of MDS (plot not shown).

References:

1. Liska, O., et al., *TFLink: an integrated gateway to access transcription factor-target gene interactions for multiple species.* Database (Oxford), 2022. **2022**.
