# Supplementary material for: Denervation alters the secretome of myofibers and thereby affects muscle stem cell lineage progression and functionality
Source: NPJ Regen Med. 2024 Mar 1;9:10. doi: 10.1038/s41536-024-00353-3 (PMC10904387; doi:10.1038/s41536-024-00353-3)
Supplement: Supplementary file 2 — Reporting Summary [file 41536_2024_353_MOESM2_ESM.pdf]

nature portfolio

Corresponding author(s): von Maltzahn, Julia

Last updated by author(s): Nov 17, 2023

Reporting Summary

Nature Portfolio wishes to improve the reproducibility of the work that we publish. This form provides structure for consistency and transparency in reporting. For further information on Nature Portfolio policies, see our Editorial Policies and the Editorial Policy Checklist.

Please do not complete any field with "not applicable" or n/a. Refer to the help text for what text to use if an item is not relevant to your study.
For final submission: please carefully check your responses for accuracy; you will not be able to make changes later.

Statistics

For all statistical analyses, confirm that the following items are present in the figure legend, table legend, main text, or Methods section.

n/a | Confirmed

- The exact sample size (n) for each experimental group/condition, given as a discrete number and unit of measurement
- A statement on whether measurements were taken from distinct samples or whether the same sample was measured repeatedly
- The statistical test(s) used AND whether they are one- or two-sided
  Only common tests should be described solely by name; describe more complex techniques in the Methods section.
- A description of all covariates tested
- A description of any assumptions or corrections, such as tests of normality and adjustment for multiple comparisons
- A full description of the statistical parameters including central tendency (e.g. means) or other basic estimates (e.g. regression coefficient) AND variation (e.g. standard deviation) or associated estimates of uncertainty (e.g. confidence intervals)
- For null hypothesis testing, the test statistic (e.g. F, t, r) with confidence intervals, effect sizes, degrees of freedom and P value noted
  Give P values as exact values whenever suitable.
- For Bayesian analysis, information on the choice of priors and Markov chain Monte Carlo settings
- For hierarchical and complex designs, identification of the appropriate level for tests and full reporting of outcomes
- Estimates of effect sizes (e.g. Cohen's d, Pearson's r), indicating how they were calculated

Our web collection on statistics for biologists contains articles on many of the points above.

Software and code

Policy information about availability of computer code

Data collection	FACSDivaTM BD Life Sciences version v8.0.1; MxPro-Mx3000P Stratagene, v4.10 Build 389, Schema 85; Zen 2, blue edition Carl Zeiss Microscopy, version 2.0.0.0
Data analysis	FlowJoTM BD Life Sciences, version v10.7.1; MxPro-Mx3000P Stratagene, v4.10 Build 389, Schema 85; Zen 2, blue edition Carl Zeiss Microscopy, version 2.0.0.0; https://www.arigobio.com/ELISA-calculator; bcl2FastQ v2.20.0.422 conversion software; CutAdapt v2.1 (-m 30 –a ADAPER-SEQUENCE); featureCounts v1.6.5;). DEseq2 package v1.26.0 ; https://david.ncifcrf.gov/tools.jsp; clusterProfiler v4.2.2; Spectronaut v13.1 (Biognosys)

For manuscripts utilizing custom algorithms or software that are central to the research but not yet described in published literature, software must be made available to editors and reviewers. We strongly encourage code deposition in a community repository (e.g. GitHub). See the Nature Portfolio guidelines for submitting code & software for further information.

Data

Policy information about availability of data

All manuscripts must include a data availability statement. This statement should provide the following information, where applicable:

- Accession codes, unique identifiers, or web links for publicly available datasets
- A description of any restrictions on data availability
- For clinical datasets or third party data, please ensure that the statement adheres to our policy

The RNA sequencing data discussed in this publication have been deposited in NCBI's Gene Expression Omnibus and are accessible through GEO Series accession numbers GSE217928 and GSE217929. Sample IDs are listed in Table 6 in the supplemental information.
The mass spectrometry proteomics data have been deposited to the ProteomeXchange Consortium via the PRIDE partner repository with the dataset identifier PXD036993.

Research involving human participants, their data, or biological material

Policy information about studies with human participants or human data. See also policy information about sex, gender (identity/presentation), and sexual orientation and race, ethnicity and racism.

Reporting on sex and gender	n/a
Reporting on race, ethnicity, or other socially relevant groupings	n/a
Population characteristics	n/a
Recruitment	n/a
Ethics oversight	n/a

Note that full information on the approval of the study protocol must also be provided in the manuscript.

Field-specific reporting

Please select the one below that is the best fit for your research. If you are not sure, read the appropriate sections before making your selection.

☒ Life sciences ☐ Behavioural & social sciences ☐ Ecological, evolutionary & environmental sciences

For a reference copy of the document with all sections, see nature.com/documents/nr-reporting-summary-flat.pdf

Life sciences study design

All studies must disclose on these points even when the disclosure is negative.

Sample size	Sample sizes for animal experiments were determined based on previous experiments analyzing similar biological aspects.
Data exclusions	we did not exclude any data.
Replication	all data are derived from biological replicates (different mice).
Randomization	The animals were randomly selected to be assigned to an experimental vs. control group.
Blinding	Blinding was not performed since this was logistically not possible: all mice need to be assigned to a dedicated group (experimental vs. control) in our mouse database. Thus, the people performing experiments will inevitably know which mouse received which treatment.

Reporting for specific materials, systems and methods

We require information from authors about some types of materials, experimental systems and methods used in many studies. Here, indicate whether each material, system or method listed is relevant to your study. If you are not sure if a list item applies to your research, read the appropriate section before selecting a response.

Materials & experimental systems

n/a	Involved in the study	
☐	☒	Antibodies
☐	☒	Eukaryotic cell lines
☒	☐	Palaeontology and archaeology
☐	☒	Animals and other organisms
☒	☐	Clinical data
☒	☐	Dual use research of concern
☒	☐	Plants

Methods

n/a	Involved in the study	
☒	☐	ChIP-seq
☐	☒	Flow cytometry
☒	☐	MRI-based neuroimaging

Antibodies

Antibodies used

Anti-eMHC Mouse-IgG1 DSHB, F1.652
Anti-Gapdh Mouse-IgG Santa Cruz, sc-365062
Anti-GFP Chicken-IgY Abcam, ab13970
Anti-Junb Rabbit-IgG Abcam, ab128878
Anti-Ki67 Rabbit-IgG Abcam, ab15580
Anti-Laminin Rabbit-IgG Sigma-Aldrich, L9393
Anti-MHC IIa Mouse-IgG1 DSHB, SC-71
Anti-MHC IIb Mouse-IgM DSHB, BF-F3
Anti-MyoD Rat-IgG2aκ Merck, MABE132
Anti-Myog Mouse-IgG1 I DSHB, F5D
Anti-Pax7 Mouse-IgG1 DSHB, Pax7
Anti-Opn Rabbit-IgG Thermo Fisher Scientific, PA5-34579
Anti-Tgfb1 Rabbit-IgG Bioss, BS-0086R
Alexa FluorTM 488 Mouse / IgG1 Thermo Fisher Scientific, A21121
Alexa FluorTM 488 Rabbit / IgG Thermo Fisher Scientific, A21206
Alexa FluorTM 488 Mouse/ IgM Thermo Fisher Scientific, A21042
Alexa FluorTM 488 Rat/ IgG Thermo Fisher Scientific, A11006
Alexa FluorTM 546 Mouse / IgG1 Thermo Fisher Scientific, A21123
Alexa FluorTM 546 Rabbit / IgG Thermo Fisher Scientific, A10040
Alexa FluorTM 647 Mouse / IgG1 Thermo Fisher Scientific, A21240
Alexa FluorTM 647 Chicken/ IgG Thermo Fisher Scientific, A21449
Alexa FluorTM 647 Rabbit / IgG Thermo Fisher Scientific, A31573
Goat anti-mouse-HRP Mouse Dako, P0447
Goat anti-rabbit-HRP Rabbit Dako, P0448
Anti-alpha-7-Integrin-AF647 Rat AbLab, R2F2, 67-0010-05
Anti-CD11B-PE Rat BD Biosciences, M1/70, 553311
Anti-CD31-PE Rat BD Biosciences, MEC 13.3, 553373
Anti-CD45-PE Rat BD Biosciences, 30-F11, 553081
Anti-Sca1-PE Rat BD Biosciences , D7, 553108

Validation

All antibodies used here were quality control tested by the manufacturer in immunofluorescent staining or immunoblot analysis. Antibodies used for Flow cytometry have been validated before in various publications.

Eukaryotic cell lines

Policy information about cell lines and Sex and Gender in Research

Cell line source(s)	n/a
Authentication	n/a
Mycoplasma contamination	Primary myoblasts were tested negative for mycoplasma contamination
Commonly misidentified lines (See ICLAC register)	n/a

Animals and other research organisms

Policy information about studies involving animals; ARRIVE guidelines recommended for reporting animal research, and Sex and Gender in Research

Laboratory animals

C57BL/6JRj, sex: male; age: 2-6 months
CAG-GFP (JAX: 003291), age: 2-6 months, male and female

Wild animals | n/a

Reporting on sex | the gender of the mice used in the respective experiment is stated in the figure legend.

Field-collected samples | n/a

Ethics oversight | All animal procedures were performed in accordance with the national regulations for animal experimentation and approved by the Thüringer Landesamt für Verbraucherschutz (license number FLI-17-015).

Note that full information on the approval of the study protocol must also be provided in the manuscript.

Plants

Seed stocks | n/a

Novel plant genotypes | n/a

Authentication | n/a

Flow Cytometry

Plots

Confirm that:

☒ The axis labels state the marker and fluorochrome used (e.g. CD4-FITC).

☒ The axis scales are clearly visible. Include numbers along axes only for bottom left plot of group (a 'group' is an analysis of identical markers).

☒ All plots are contour plots with outliers or pseudocolor plots.

☒ A numerical value for number of cells or percentage (with statistics) is provided.

Methodology

Sample preparation

Single myofiber analysis
Single myofiber isolation was performed as described earlier [21]. Briefly, extensor digitorum longus (EDL) muscles were carefully excised from tendon to tendon and digested in a 0.2 % collagenase (w/v) solution for 60 – 90 min at 37 °C, thereby eliminating cells that are not under the basal lamina. Single myofibers were obtained by carefully triturating the digested muscle with a horse serum coated glass Pasteur pipet with a broken-off heat polished tip. 50 – 100 single, non-contracted myofibers were used for each experimental condition. For RNA sequencing, myofibers were directly transferred to TRIzol reagent for RNA isolation. For immunofluorescence staining, myofibers were either immediately fixed after isolation or cultured for up to 72 h at 37 °C and 5 % CO2 before fixation and staining. Myofiber supernatants were collected from cultured myofibers at the respective times and immediately used in a 1:1 ratio with fresh culture medium.
FACS-isolation of MuSCs
Hind limb muscles were harvested, minced with scissors and digested enzymatically with collagenase B (10 mg/ ml, Roche) and dispase II (4 mg/ ml, Roche) in PBS at 37 °C for 30 min under trituration every 10 min. The suspension was mixed with growth medium (Ham's F-10 Nutrient Mix + 20 % FBS + 1x P/S + 2.5 ng/ml bFGF), filtered through a 74-µm pore size strainer (Corning Life Sciences), spun down and resuspended in FACS buffer (PBS with 2 % FBS) with the respective antibodies listed in Table 4 in the supplemental information. After 15 min incubation on ice, samples were spun down, resuspended in FACS buffer and filtered through a 35-µm nylon mesh (Corning Life Sciences) before 1 mM SYTOXTM Blue dead cell stain was added 1:1000. MuSCs were identified as α7-integrin+ - Sca-1- - CD11b- - CD31- - CD45- - Sytox- using a FACSAria III with Diva software v8.0.1 (BD Life Sciences). For MuSCs from CAG-GFP mice cells were additionally sorted for GFP signal.
Proteomic analyses
MuSCs were FACS-sorted into 2x lysis buffer and processed for MS-based proteome analysis as described [33]. A minimal cell number of approximately 100.000 cells per sample was used for each replicate. Briefly, samples were lysed and homogenized, boiled at 95 °C for 10 min and sonicated at 20 °C. After incubation with dithiothreitol (final concentration 10 mM, 45 °C) and addition of iodoacetamide (final concentration 15 mM, RT in the dark), samples were incubated with eight volumes ice-cold 100 % acetone at -20 °C over night before washing twice with 80 % ice-cold acetone. The pellet was air-dried, digested (3 M Urea and 100 mM HEPES, pH=8) and sonicated. Addition of Lys-C (final concentration 0.05 µg/µl, 4 h at 37 °C) was followed by 1:1 dilution with HPLC water and Trypsin digestion (1:100 enzyme: protein ratio) over night at 37 °C. Samples were acidified by addition of 10 % trifluoroacetic acid to a final of 1 % and desalted using a Waters Oasis HLB µElution Plate 30 µm (Waters, #186001828BA) according to the manufacturer`s instruction. Eluted samples were dried in a SpeedVac at 45 °C before reconstitution to 1 µg/µl in 10 µl of buffer (0.1 % formic acid in 5 % acetonitrile in HPLC water) and homogenization. The iRT kit was used as an internal control in a dilution recommended by the manufacturer. Peptides were separated using the nanoAcquity UPLC MClass system (Waters) with the Proxeon nanospray source. For data acquisition and processing of the raw data Xcalibur v4.0, Tune v2.1 (Thermo Fisher) and Spectronaut v13.1 (Biognosys) were used with the

	default settings.
Instrument	sorting: BD FACSAria III Fusion cell sorter
Software	FACSDivaTM BD Life Sciences version v8.0.1
Cell population abundance	We determined post-sort purities when pooled samples were collected.
Gating strategy	The gating strategies are given as supplemental figures. To identify dead cells in the FSC/SSC population SytoxBlue was added to the samples and based on this, the FSC/SSC was set to exclude dead cells. Isotype controls and/or fluorescence-minus controls were used to define negative cell populations.

☒ Tick this box to confirm that a figure exemplifying the gating strategy is provided in the Supplementary Information.

This checklist template is licensed under a Creative Commons Attribution 4.0 International License, which permits use, sharing, adaptation, distribution and reproduction in any medium or format, as long as you give appropriate credit to the original author(s) and the source, provide a link to the Creative Commons license, and indicate if changes were made. The images or other third party material in this article are included in the article's Creative Commons license, unless indicated otherwise in a credit line to the material. If material is not included in the article's Creative Commons license and your intended use is not permitted by statutory regulation or exceeds the permitted use, you will need to obtain permission directly from the copyright holder. To view a copy of this license, visit http://creativecommons.org/licenses/by/4.0/
